# Particle Detection and Characterization for Biopharmaceutical Applications: Current Principles of Established and Alternative Techniques

**DOI:** 10.3390/pharmaceutics12111112

**Published:** 2020-11-19

**Authors:** Julia Gross-Rother, Michaela Blech, Eduard Preis, Udo Bakowsky, Patrick Garidel

**Affiliations:** 1 Department of Pharmaceutics and Biopharmaceutics, University of Marburg, Robert-Koch-Str. 4, 35037 Marburg, Germany; julia.gross-rother@boehringer-ingelheim.com (J.G.-R.); eduard.preis@pharmazie.uni-marburg.de (E.P.); 2 Innovation Unit, PDB, Boehringer Ingelheim Pharma GmbH & Co. KG, 88397 Biberach an der Riss, Germany; michaela.blech@boehringer-ingelheim.com

**Keywords:** particle detection, particle characterization, emerging particle techniques, protein aggregates, particle quantification, biopharmaceuticals

## Abstract

Detection and characterization of particles in the visible and subvisible size range is critical in many fields of industrial research. Commercial particle analysis systems have proliferated over the last decade. Despite that growth, most systems continue to be based on well-established principles, and only a handful of new approaches have emerged. Identifying the right particle-analysis approach remains a challenge in research and development. The choice depends on each individual application, the sample, and the information the operator needs to obtain. In biopharmaceutical applications, particle analysis decisions must take product safety, product quality, and regulatory requirements into account. Biopharmaceutical process samples and formulations are dynamic, polydisperse, and very susceptible to chemical and physical degradation: improperly handled product can degrade, becoming inactive or in specific cases immunogenic. This article reviews current methods for detecting, analyzing, and characterizing particles in the biopharmaceutical context. The first part of our article represents an overview about current particle detection and characterization principles, which are in part the base of the emerging techniques. It is very important to understand the measuring principle, in order to be adequately able to judge the outcome of the used assay. Typical principles used in all application fields, including particle–light interactions, the Coulter principle, suspended microchannel resonators, sedimentation processes, and further separation principles, are summarized to illustrate their potentials and limitations considering the investigated samples. In the second part, we describe potential technical approaches for biopharmaceutical particle analysis as some promising techniques, such as nanoparticle tracking analysis (NTA), micro flow imaging (MFI), tunable resistive pulse sensing (TRPS), flow cytometry, and the space- and time-resolved extinction profile (STEP^®^) technology.

## 1. Introduction

Particles and particulate matter have enormous influence on daily life. Detecting and characterizing particles and their properties (especially in suspensions and colloids), in the visible and subvisible size ranges, is essential in fields, like electronics, pollution control, cosmetics, and biopharmaceuticals (e.g., therapeutic proteins). While the definition of “particle” is very ambiguous, we will be referring to subvisible macromolecules and aggregates with diameters of a few nanometers up to about 100 micrometers. These nanosuspensions have attracted growing interest in medicine and biopharmaceuticals [1]. Examples include poly (lactic-*co*-glycolic acid) (PLGA) particles or liposomes as drug carriers or gold nanoparticles used for analytical purposes [2,3,4,5,6]. Perhaps, understanding the properties of naturally occurring particles, like exosomes and protein complexes, is even more fundamental to elucidating their roles in health and disease [7,8,9,10,11,12]. The topic is crucial for all kinds of biopharmaceuticals and the techniques may be also suitable for other biomolecules, e.g., DNA or virus. At this point, however, we will focus especially on therapeutic proteins and their requirements and challenges. In biopharmaceutical protein formulations, especially in parenteral formulations, particulate contaminants like protein aggregates or extrinsic particles, such as residual glass traces, are a safety and quality concern [13,14,15]. If undetected particle contaminations reach patients, they can contribute to vascular occlusion, pulmonary embolism, or induce immunogenic reactions [13,16,17]. Ahmadi et al. demonstrated that the presence of even small amounts of protein particles may have strong immunogenic potential [18]. Freitag et al. further showed how immune reactions can depend on the particle/aggregate type [17]. Such studies highlight the need for highly sensitive, accurate detection and characterization techniques to ensure safety and prevent immunogenic reactions [17,18,19,20,21,22,23]. Identifying and characterizing particles in the laboratory can help both assess their risk to the patient and trace their origins [24]. Ultimately, the biopharmaceutical particle analyst’s goal is understanding particle origin and behavior to keep particles from forming, or detect and remove them in case they have been observed.

Together, new insights, increasing knowledge, and rapid technical development have led regulatory authorities to adjust pharmaceutical approval and quality regulations regarding particulates, e.g., United States Pharmacopeia (USP) <787> or <788> for parenteral formulations [25,26]. Both USPs set limits for particle sizes and their respective concentrations and define the detection protocol and the instrument based on the current scientific and technical knowledge. To comply with regulations like these, companies have to be prepared and stay up-to-date. USP<1787>, for example, describes methods for particle analysis, embracing methods both new (e.g., flow microscopy imaging, FMI) and established (e.g., light obscuration, LO) [26,27].

The analyst, however, must choose the technologies most appropriate to the colloid solution under investigation. There are two main challenges:

(1) the *nature and the properties of the particle sample* (the particle and suspension medium) itself
(2) the underlying *measurement principle of the technique*, with its sample-preparation requirements

The literature on principles, methods, and challenges in biopharmaceutical particle detection has skyrocketed in recent years. Thus, this article will (i) take a closer look at the relevant range of current particle analysis principles and techniques, (ii) illustrate the challenges posed by biopharmaceutical protein samples, and (iii) give an overview of some of the most promising new and alternative approaches for particle analysis of therapeutic protein samples.

## 2. Particle Properties and Current Technical State

### 2.1. Common Properties of Particle Suspensions

**Figure 1:**
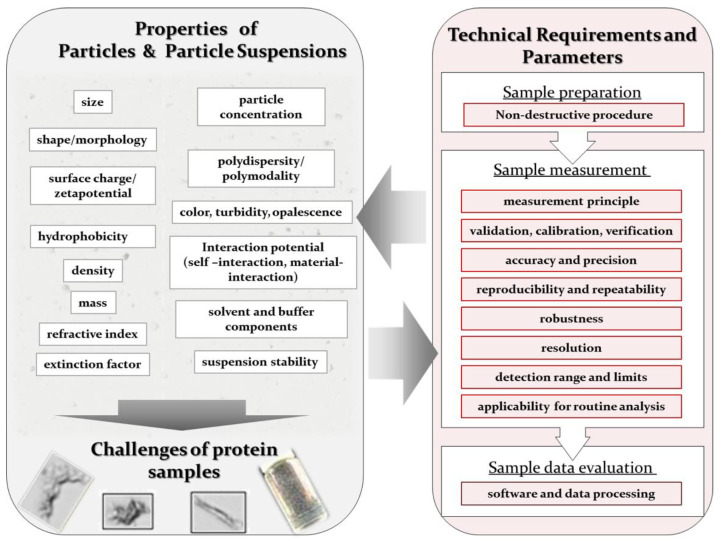
Particle properties and resulting technical requirements. The left part summarizes some of the most investigated properties of particles. The right part monitors the analytic workflow and points out technical parameters essential for particle detection techniques. Both parts need to be considered to identify a suitable technique and to interpret the results of an analysis.

All particle detection and characterization techniques are based on, and highly sensitive to, the physicochemical properties of the investigated sample and sample components [28]. For that reason, considerations of the nature and properties of the sample are highly important to identify a suitable technique. For result interpretation it is key to understand interdependencies in nanosuspensions as illustrated in Figure 1. In case of particle suspensions, one needs to consider the properties of each single particle as well as the properties of the overall suspension. An overview about frequently investigated and considered sample properties is given in Figure 1 (left).

In general, each *individual particle* in a suspension has its own physical and chemical properties. The analyst must consider both the collective and individual characteristics when developing a strategy. Typical particle properties of interest include nature (e.g., foreign, proteinaceous), size, morphology or shape, surface charge or zeta potential, hydrophobicity, homogeneity, and reversibility [3,28,29,30,31,32,33,34]. Depending on the technique used, some properties may need to be defined before the property of primary interest can be determined. For example, the refractive index must be known to make sense of light-scattering techniques; transparency must be known when applying imaging techniques; material properties like density, are needed to apply sedimentation techniques [35,36,37]. The determination of such prerequisite properties, and considering these properties during data analysis, in particular optical properties, can be challenging [35,36,38]. Distinguishing between particles of different materials is important for tracing their origins and influence on the product [39,40,41]. Many analytical techniques, however, measure only *overall suspension properties (ensemble-properties)*, e.g., the particle concentrations, particle size distributions (PSD), polydispersity, viscosity, color, or turbidity/opalescence of the whole particle population [42,43,44,45,46]. Typical ensemble PSD evaluations are intensity weighted, volume weighted, surface weighted, or number weighted. To develop a more detailed view, the fundamentals of colloid science should be applied [1,42]: particle–particle interactions (protein–protein-association) as well as their nature (reversible/irreversible, covalent/electrostatic) and particle–solvent interactions play essential roles [46,47,48,49,50,51]. The analyses mentioned above produce better stability analyses and shelf-life estimates [46,52].

In case the particles are suspended in a fluid, the properties of the fluid and its components (e.g., salt ions or sugars) must also be taken into account to gauge shielding effects or volume exclusion [49,53,54,55,56]. In fact, the suspension needs to be seen as a dynamic continuum [1,45,57].

A general classification of particles is hardly achieved considering the variety of properties and, to date, no standardized, uniform categorization system has been established. In general, particles can be classified according to each property, e.g., by size in subvisible and visible particles, by shape in spherical and nonspherical or by material in foreign and proteinaceous [24,31,58,59,60]. In pharmaceuticals an often used classification differentiates extrinsic, intrinsic, or inherent particle matter.

Out of the universe of possible properties, we generally concentrate on four that are especially important: particle size and size distribution (for polydisperse/polymodal suspensions), concentration, shape, and surface charge. The most important parameter is particle size and size distribution related to the particle concentration. This is partly because of the range of approaches and orthogonal principles for determining particle size. Furthermore, increasing particle sizes can be directly correlated with increasing aggregation/agglomeration or suspension instability [61,62,63,64,65,66]. In addition, that particle size affects many aspects of particle behavior [19,30,67,68,69,70,71,72]. Haskell, for example, showed that a formulation’s in vivo performance can be regulated by controlling particle size [73]. Other studies have demonstrated a correlation between size and cellular uptake efficiency of nanoparticular matter [30,68,73]. Larger particles increase the incidence of vascular occlusion, and so must be excluded from parenteral preparations [74,75,76]. Based on the Derjaguin, Landau, Verwey, and Overbeek (DLVO) theory, which explains charged-particle aggregation in an aqueous dispersion, colloidal stability is defined by attractive and repulsive noncovalent interactions between the particles [46,77,78,79]. This theory enables researchers to estimate a formulation’s physical stability based on particle size and surface charge. In practice, supportive parameters, like the second osmotic virial coefficient B_22_, the bulk diffusion/restricted diffusion interaction coefficient k_D_ or the zeta potential help to estimate colloidal stability [46,80].

In contrast to the description by size determinations, particle concentration determinations permit *quantitative* characterization of the system [81]. Spatial and time limitations prevent robust real-time detection of every particle at every moment. Quantification nevertheless remains an option for comparing samples and observing small changes to the system [73]. In addition, concentration considerations are essential for estimating viscosity and predicting the behavior of highly concentrated liquid formulations (HCLFs) for technical applications [82,83,84].

One need only review medicine authority regulations to acknowledge the importance of size and concentration determinations. Perhaps because particle size and concentration can be easily stated, limits on their values figure prominently in regulations. For example, USP<787> sets limits for light-obscuration (LO) determined concentrations of subvisible particles with diameters of 10 µm (≤6000 particles per container) to 25 µm (≤600 particles per container) in parenterals [25].

In general, it is always important to remember that the value measured for any property will depend on the analytic technique and protocol used (Figure 1, right). There is no unique property that can be described by different methods in the same way. Take the determination of something as fundamental as particle size, for example. Microscopy is the only visual and direct technique/principle to determine particle size, and even here, sample preparation steps, such as drying on microscopy carrier, can impair the results by producing artefacts [16,34,85,86,87]. In the absence of direct size determination approaches, most particle-by-particle measurements and higher throughput methods rely on the concept of *equivalent particle diameter* (EPD) [16]. The concept posits a spherical particle that would produce the same read-outs as the presumably more complex, nonspherical, sample. The precise definition of EPD depends on both, the measurement principle and the physical particle attribute measured, as well as the final data processing and evaluation [16]. For example, the *hydrodynamic diameter*, as determined by dynamic light scattering (DLS), is not the same as the diameter measured directly by microscopy and imaging techniques. Direct imaging techniques can measure multiple parameters, adding values like aspect ratio and the average diameter value [88,89,90]. Few particles are actually spherical, so estimating the distribution of shapes is important. A figure for particle size distribution (PSD) refines the picture for polydisperse suspensions, where a single average-diameter figure gives a misleading description. The PSD includes parameters for distribution ranges (e.g., D10 and D90) to the mean equivalent [16,34].

Finally, particles, in particular biological particles, are often heterogeneous, highly dynamic, and extremely sensitive to small environmental changes. Sample preparation can also change the results of particle size measurements. Buffer exchanges alter hydrodynamic diameters. Dilutions redissolve agglomerates. Harsh preparation conditions can force agglomeration and affect results [91,92,93].

Clearly, detailed knowledge of the sample’s properties is required when selecting the most suitable measurement principles and techniques. This information helps to guide preparation, avoid interactions with equipment and excipients, and set suitable measurement parameters. For data evaluation and interpretation, estimations concerning signal fluctuations and inaccuracies caused by the sample can be made and technical limits are evaluated.

### 2.2. Specific Properties and Challenges of Biopharmaceutical Protein Samples

Biologicals, such as therapeutic proteins, are highly challenging samples. Monomers of monoclonal antibodies (mABs), for example, show monomeric diameter in the low nanometer range (approx. 10 nm) and a nonspherical Y-shape [94]. Indeed, the size difference between mAB monomers and the surrounding solvent molecules is large enough that a mAB suspension can be handled as colloid suspension [42]. In this article, “protein particle” means any protein-containing assembly, independent of nature and structure, from monomers to aggregates or associates that range from dimers to extremely large multimeric units with diameters rising above 100 µm diameters [95,96,97,98]. Therefore, any investigation of such samples should cover the whole range from 10 nm to 100 µm. The term “aggregate” applies to protein assemblies of higher molecular order formed by at least partially unfolded monomers [47,99].

In general, protein samples, in particular stressed protein samples that contain aggregates and associates over a wide size range, are extremely challenging for following reasons:

(i) **Specific individual particle properties.** Some specific particle properties, especially optical and material properties, can be hard to determine. Each particle could potentially have distinct and individual properties. For example, the refractive index of a protein suspension is estimated to be between 1.3 and 1.6 [87,100,101]. In a recent study, using comparative light transmission and scattering measurements, Zölls et al. determined a refractive index of 1.41 for their investigated monoclonal antibody aggregates [35]. Another example is the particle density. In 1979, Squire and Himmel found the density of protein’s mass density to be about 1.37 g/cm^3^ [102]. In 2004, Fischer et al. summarized three theoretical and two experimental studies and suggested that protein density ranges between 1.22 and 1.43 g/cm^3^ [103]. They formulated a general expression for estimating a protein’s density based on its molecular weight [103]:

(1) $$\rho \left(M\right)=\;\left[1.41+0.145\;e^{-\;\frac{M}{13}}\right]\;g/{\mathrm{cm}}^{3}$$

where *ρ* is the density of a molecule of molecular weight *M* in kiloDaltons (kDa).

Thus, a 150 kDa monoclonal antibody would have an estimated density of 1.41 g/cm^3^. As $e^{-\frac{M}{\;13}}$ becomes vanishingly small as *M* increases, the densities of high-molecular-weight proteins remain almost constant at this value. It is not clear, however, whether the Fischer estimation applies to suspensions that include aggregates with variations of the structure (densely packed vs. loosely packed). Several approaches have been published for the experimental determination of densities, e.g., by suspended microchannel resonators or density gradient centrifugation [38,104,105].

(ii) Protein suspension **composition and properties**. A protein suspension is highly heterogeneous and polydisperse, with particle sizes that can range up to six orders of magnitude [95,96,97,98]. As these complex structures have multiple degradation and/or aggregation pathways, the suspension may contain innumerable variants of monomers (native, partially unfolded, unfolded), associates, and aggregates [63,99]. The result can be chemical or physical aggregates with altered chemical or physical properties in the size ranges similar to the target species but with a change of conformation [63,99,106,107,108,109,110]. Another challenge is the suspension composition with a high concentration of small monomers, dimers, and oligomers and low concentrations of large associates and aggregates. Some exemplary issues that occur because of populations of high concentration are blockage of the system (pore blockage), reduced chances of single particle detection while overlaying signals are maximized, or the occurrence of multiple scattering causing false results. In contrast, low concentrations may only give low signals and populations with low concentrations might not be detectable in the presence of populations with larger concentrations. This is particularly described for DLS measurements. The ratio relationship between concentration/size and light-scattering intensity is nonlinear, making light-scattering techniques challenging for such suspensions. In consequence, the scattering intensity of high concentrations of small particles are interfered by small amounts of larger particles and the populations are hardly distinguishable. This was nicely shown by Karow et al. [83]. In general, along the same line of reasoning, bulk measurement methods (i.e., those that do not separate different species for individual measurement) also appear problematic.

(iii) Thermodynamic **equilibrium and protein aggregation.** A protein suspension is a highly complex, dynamic, and transient system. Multidomain proteins, like monoclonal antibodies, have a specific functional structure (native state) [94]. Within their structural frame envelope, however, the conformation is highly dynamic, characterized by variable intramolecular interactions. These may lead to the transient formation of associates or aggregates, which are thermodynamically unstable but kinetically stable if an energy barrier is high enough [61]. In addition, a protein suspension is highly sensitive to changes in macro- and micro-environment [65,111,112,113]. Sample preparation and measurement conditions may cause destructive effects and trigger aggregation pathways that lead to “false”, misleading results. Techniques with harsh preparation conditions, (e.g., heating, dilution, or changes in buffer compositions) alter the sample properties [91,92,93]. Protein aggregation processes have been a hot topic over the last decades and are still hardly understood. Aggregation mechanisms and pathways are initiated and influenced by a great variety of factors and the stabilization of proteins and the prevention of protein aggregation are still challenging [42,53,62,63,99,114,115,116,117,118,119,120,121,122].

(iv) Crowding and aggregation effects in **high concentrated liquid formulations (HCLF).** Subcutaneous administration of (bio-) pharmaceutics opens several advantages compared to other administration ways [123,124]. One limitation is the injected volume being below 1–2 mL. For that reason, the use of HCLFs is a possible application, but leads also to new challenges. Such challenges are aggregation due to molecular crowding effects, high viscosities, and the lack of suitable analytical techniques [46,106,119,125,126,127,128,129].

(v) The increased potential to interact with the **packaging material**. Proteins exhibit a heterogeneous surface with charged and hydrophobic patches. This leads to an increased potential to interact with different kinds of packaging material, e.g., glass syringes, stainless steel, tungsten, and leachables causing aggregation [130,131,132]. For that reason, extrinsic particles originating from such contact material in protein suspensions have to be identified and discriminated as well. One popular example is silicon oil. Siliconization is necessary for better mechanics of syringe performances. Several studies investigated the effects of silicon oil in protein formulations, such as the risk of aggregation initiation and immunogenic risks [39,40,133,134]. The identification, differentiation, and characterization of silicon oil droplets is realized by a few techniques such as MFI or SMR [135].

(vi) The lack of appropriate consistent **protein (calibration) standards** that mimic the protein properties. Assays and technologies that employ a calibration standard pose a further challenge. In most cases, the used standards employ spherical particles of polystyrene or silica. The physicochemical properties, especially the refractive index of these particles, however, differ significantly from those of the protein particles under investigation. To be more reliable, these techniques require calibration-standard particles whose properties are closer to those of the target protein. Over the last decade various approaches have been described, e.g., by Ripple et al. from the National Institute of Standards and Technology, which used particles of ethylene tetrafluoroethylene (ETFE) [97,136,137]. Another approach is to fashion particles of specified size by cross-linking proteins, such as the albumin (e.g., BSA) particles produced by Micromod Partikeltechnologie [83]. Matching property of the refractive index is far from being the only issue. For analytical data evaluation, most calculations assume spherical particles; but sample particles come in innumerable shapes. Thus, developing a generally applicable calibration standard remains difficult.

The analysis of protein samples and particles will remain highly challenging. As protein particles’ microscopic and macroscopic properties are so variable (Figure 1), no single analytical method can reliably capture all of the relevant properties at all time scales. Every technique applied on its own will give results with a certain bias and, thus, any single technique will retain some inherent ambiguity. Protein samples should therefore be tested using a toolbox of orthogonal methods, and the need for more suitable techniques for protein particle detection and characterization techniques remains.

### 2.3. Particle Detection and Characterization in Biopharmaceutics

**Figure 2:**
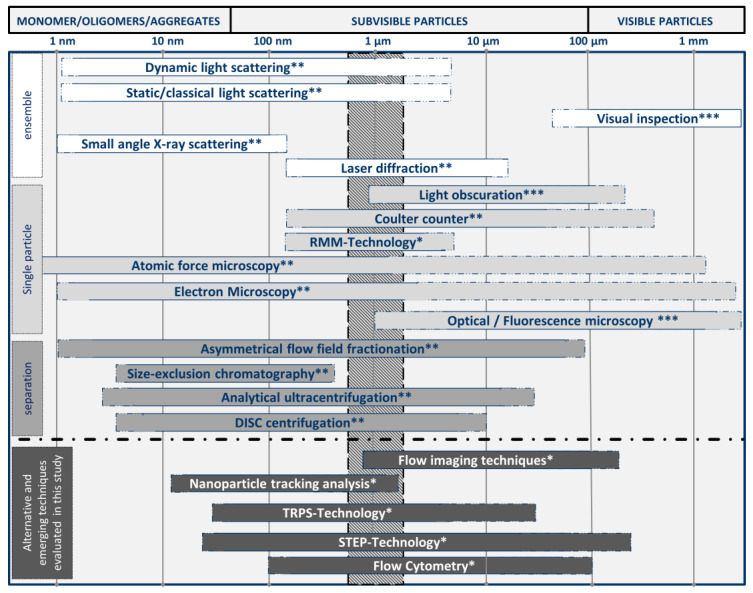
Overview particle detection techniques. Established (top part) and emerging (lower part) particle detection techniques are summarized according to their size measurement range. Their applicable size range is illustrated by the logarithmic scale at the top and the detection gap around 1 µm is highlighted by a grey bar. Further the techniques are classified into ensemble, single particle, and separation techniques. Stars indicate their current state: * emerging technique; ** established and often used techniques for pharmaceutical applications; *** techniques mentioned in US Pharmacopeia. RMM = Resonant Mass Measurement, DISC = Disc based centrifugation, TRPS = Tunable Resistive Pulse Sensing, STEP = Space- and Time-resolved Extinction Profile.

A broad range of techniques is available and needed for analyzing and characterizing the huge range of particle properties, particularly in biopharmaceutical applications. A selection of established and emerging techniques for biopharmaceutics is given in Figure 2. As illustrated, these techniques can be classified by the size range of the particles for which they are particularly suited or by their measurement type as ensemble (or bulk), single-particle, and separation-based measurement techniques (Figure 2) [73,138]:

**Ensemble (or bulk)** measurement techniques detect a single overall signal from the particle suspension and display average properties of all particles [73]. Established examples for ensemble measurement techniques are mostly optical based techniques, such as dynamic light scattering (DLS), static light scattering (SLS), light diffraction, and turbidity determinations. These types of measurements are sufficient for homogenous monomodal and low-polydispersity suspensions. Ensemble measurements of more polydisperse suspensions might yield low resolution, biased data, or yield incomplete descriptions of the particle population and particle properties. As these methods do not measure any particles directly, they cannot produce reliable number-based particle size distributions. The calculation of number-weighted or volume-weighted particle size distributions gives an estimate only as it is based on the assumption of spherical particles. In terms of DLS, a thorough description of the differences between the mentioned distributions and the underlying calculations was provided by Stetefeld et al. [139]. In consequence, ensemble measurements result in estimated particle concentrations only.

In contrast, **single-particle** measurements detect, characterize, and report properties particle by particle [73]. Common established methods include microscopic and imaging techniques, such as optical, infrared or fluorescence microscopy, transmission electron microscopy (TEM), and atomic force microscopy (AFM). Further prominent examples are coulter counters and the suspended microchannel resonant mass measurement (RMM). Light obscuration (LO) is a particularly important technique in this category. It is a listed pharmacopoeial method measuring subvisible particles in parenterals (USP<787>, USP<788>) [25,26]. Two other methods are recommended for measuring particles in other size ranges: optical microscopy for nonspherical particles greater 1 µm (USP<776>, USP<788>) and visual inspection of visible particles (USP<790>) [140,141]. Direct characterization of every particle in the suspension is not really practicable because of greater time requirements; however, direct quantification leads to more reliable number-weighted PSDs and more precise extrapolated particle concentrations. A disadvantage is that the concentration ranges are mostly narrow and limited as, in consequence, the sample concentration has to be adjusted, e.g., by dilution or long measurement times and high sample volumes.

**Separation**-based techniques use various principles to separate particles before or during the analysis. This category includes centrifugation techniques—such as differential centrifugal sedimentation (DCS) or analytical ultracentrifugal (AUC) filtration—size exclusion chromatography (SEC) and asymmetric flow-field flow fractionation (AF4). These measurement approaches overcome the drawbacks of ensemble measurement principles and are often combined with the latter, e.g., by SEC-MALLS.

Emerging techniques (discussed comprehensively in Section 4 of this article) show a general trend towards single-particle measurements (Figure 2, bottom). These approaches may show improved accuracy, repeatability, resolution, less bias impaired, and throughput than established techniques—especially in multimodal and polydisperse samples, for which resolution is particularly important.

Despite these improvements, the fact remains that there is no single, universal particle detection method that can fulfill all requirements in all applications. None of the available techniques can cover the full range of sizes over the periods of time needed to full describe protein suspensions and aggregation processes [42,63,117,142,143]. Some techniques require dilutions that could change the characteristics of the sample. Additionally, only a few (RMM, MFI) can discriminate among particles of different types [135]. Other methods demand sample volumes that are immense compared to the therapeutic dosage volume. High-throughput analyses are highly desirable in today’s biopharmaceutical production environment; so far, however, few standard particle-detection methods come equipped to meet this demand. Methods that depend on user-defined settings or manual operations make comparability difficult [144,145]. These and other limitations highlight the need for a more general, more robust approach, a need that will become more acute as process developers continue the trend to highly concentrated liquid formulations (HCLF).

## 3. Principles of Common Particle Analysis, Detection, and Characterization Methods

Commercially available particle analysis instruments are based on a few basic physical principles [16,28,146]. Some of them are based on natural phenomena well known for hundreds of years; others, on principles that have emerged over the last decades. Here, we will briefly describe them and summarize their application, advantages, and shortcomings.

### 3.1. Particle–Light Interaction

**Figure 3:**
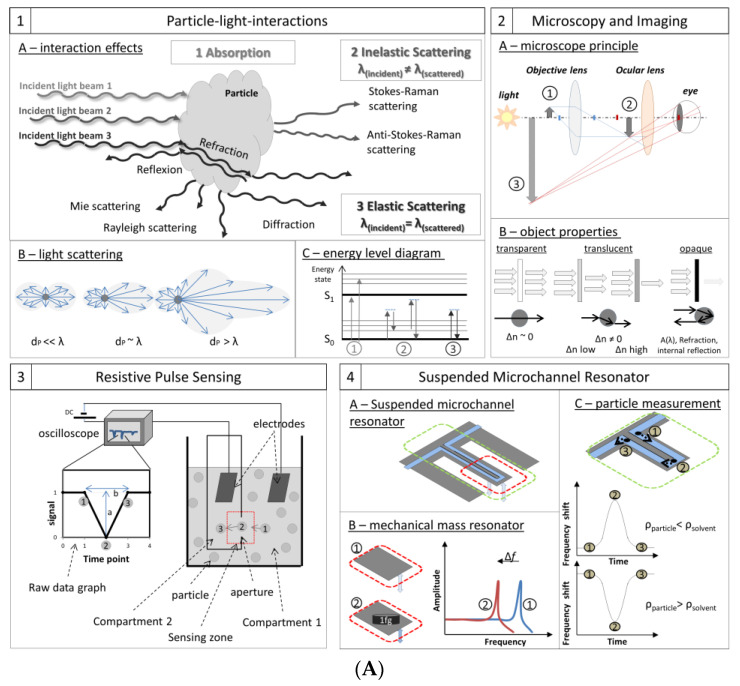

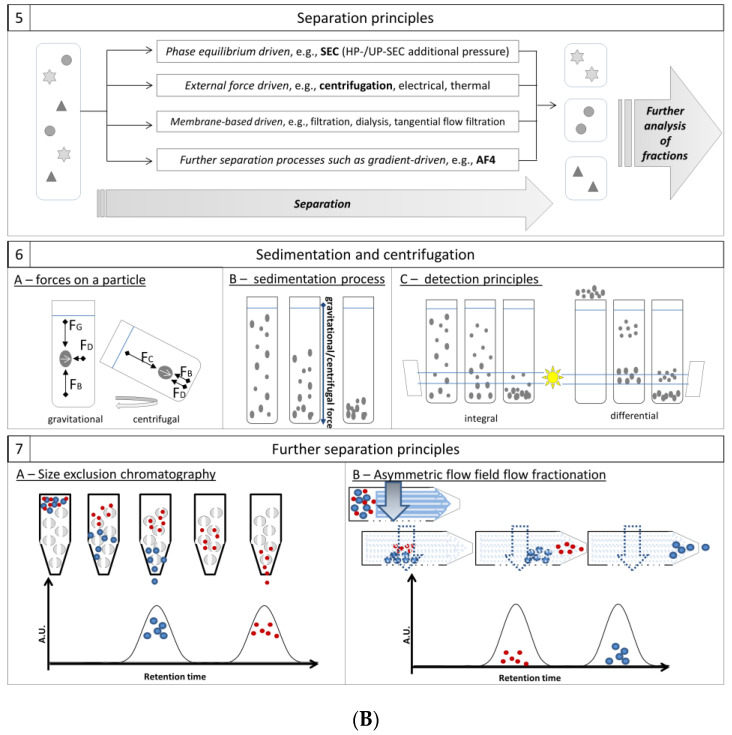
(**A**) Schematic illustration of important particle detection principles. The major principles of particle detection techniques, particle–light interactions (**1**), microscopy and imaging (**2**), resistive pulse sensing or Coulter principle (**3**), and suspended microchannel resonators (**4**) are summarized. (**1A**) There are different effects of particle–light interaction after an incident light beam encounters a particle. (**1B**) Based on the Mie theory light scattering intensities in a spatial cross area are characteristic for specific particle sizes (d_P_) depending on the wavelengths of the incident light beam. (**1C**) The final effects are determined by the energetic state the photon causes in the molecule. (**2A**) Microscopic magnification of tiny objects is based on a complex optical system. A reel image 2 of the object 1 is achieved by the objective lenses. This image is subsequently magnified by the ocular lenses to the virtual image 3. (**2B**) Depending on the optical properties of particles, e.g., the refractive index and its difference to the surrounding fluid, these particles show different effects and behavior by light interactions: transparent, translucent, or opaque. (**3**) A Coulter counter consists of two compartments filled with an electrolyte and connected by a small pore. In each compartment, an electrode is present, and a direct current is applied. Adding the particle to one compartment, they pass (1→2→3) the pore inducing a resistive pulse in the applied current. This pulse is monitored and gives information about the size by its amplitude, *a*, and its shape by the blockage duration, *b*. Furthermore, the pulse event frequency is correlated to the particle concentration. (**4A**) Schematic representation of a suspended microchannel resonator (SMR). (**4B**) Already the addition of 1 femtogram causes a frequency shift. (**4C**) If particles pass the microchannel the frequency changes. Depending on the density difference between particle and fluid, peaks appear either positive or negative. (**B**) Schematic illustration of important particle detection principles (i.e., general separation principles (**5**), sedimentation and centrifugation (**6**), and further established separation techniques (**7**)). (**5**) Overview about the general separation process and typical principles. (**6A**) The phenomenon of sedimentation/centrifugation is caused by the balance of three forces: *F_G_* gravity force, *F_D_* drag force, and *F_B_* buoyancy force. For centrifugal sedimentation the gravity force is replaced by the centrifugal force *F_C_*. (**6B**) During naturally occurring sedimentation processes, particles in suspension settle down (gravitational or centrifugal) in the direction of the gravitational or centrifugal force with a sedimentation velocity depending on their particle size and density. (**6C**) The sedimentation can either be detected and analyzed by an integral method (left) or by a differential method (right). (**7A**) The separation principle of size exclusion chromatography based on the molecular size is illustrated. The sample passes a resin containing pores and depending on particle size they either enter this pore leading to a delayed retention compared to larger particles that just flow through without entering. (**7B**) The separation principle of asymmetric flow-field flow fractionation based on hydrodynamic diffusion behavior is illustrated. The sample passes a channel with pores moving in a vertical flow. The application of a cross flow causes a separation of smaller particles that move faster in the parabolic flow and the larger particles closer to the channel wall.

Light interacts with matter in a variety of ways, primarily absorption (where energy is transferred from the light to the particle), elastic scattering (including phenomena like Mie and Rayleigh scattering, in which photon energy and wavelength are conserved), and inelastic scattering (including Stokes–Raman and anti-Stoke–Raman scattering, in which the energy and light wavelength change) (Figure 3(A1)). More detailed information and mathematical calculations can be found elsewhere [147,148,149,150,151,152,153,154,155,156,157,158].

#### 3.1.1. Principles

**Elastic Scattering and Mie Theory.** Light scattering, especially elastic light scattering, is the process exploited most often in instruments to show particle size and shape. Light scattering is commonly the sum of the physical processes of reflection, refraction, and diffraction.

From a molecular perspective, incoming photons interact with electrons in the particle, increasing their energies within the ground state (S_0_). This creates charge separations and oscillation induced dipole moments. These oscillations trigger the emission of photons. In short, light scattering can be seen as fast absorption and instantaneous emission of photons [159,160,161].

**Rayleigh scattering and Mie scattering.** Rayleigh scattering (by particles much smaller than the photon wavelength, *d_P_* << *λ*) and Mie scattering (by spherical particles with diameters about the same as the wavelength, *d_P_* ≈ *λ*) are elastic processes: incident and scattered photons have the same wavelengths and energies, and the spatial distribution of scattered photons is predictable (Figure 3(A1B)). Rayleigh scattering disperses light symmetrically in every direction. As the ratio of particle diameter to wavelength increases, scattered light waves interfere. Interference phenomena result in Mie scattering, in which most of the light scatters ahead of the incident beam, making it appear that the light is bending around the particle. In this regime, spherical particles produce a higher ratio of forward-scattered to back-scattered light than coiled or rod-shaped particles [159,160,161]. For evaluation, the Mie theory mathematically describes the resulting scatter patterns as a function of incident wavelength, refractive index, and particle size and shape. Consequently, scatter patterns can be analyzed to yield valuable information about the scattering particles.

**Diffraction.** Diffraction is one of the physical processes causing scattering effects. The light bending around an object (an edge, a particle, or a hole) creates patterns that depend on the light’s wavelength and the size of the object, which makes it particularly useful for detecting and characterizing particles. In particular laser diffraction studies are applied [158].

*QELS.* A third approach connected to elastic scattering for indirect size determination is **quasi-elastic scattering** (QELS) or **dynamic light scattering** (DLS). In this case scattered light fluctuations over time are used to observe Brownian motion of particles and determine their diffusion coefficient. The size of the particle can be calculated from the Stokes–Einstein relation (given below, Equation (13)), which describes how spherical particles diffuse through low-Reynolds-number liquids [162].

**Inelastic scattering or Raman scattering.** In inelastic light scattering, energy is exchanged between the photon and the matter, so that incident and scattered frequencies of the light differ. Similar to elastic scattering the photons are first absorbed. The incoming photon excites an electron to a higher virtual energy state but within the same energy level causing specific vibration within the molecule. Subsequently, the electron relaxes to an energy level that is different from the initial level, so the frequency of the emitted light is different from the incident photon, and is characteristic for atoms or molecules (Figure 3(A1C2)). Raman spectroscopy is the most common inelastic scattering technique for the investigation of structural properties.

**Absorption.** Light absorption occurs when incident photons have the specific energy/wavelength to excite the electron in a higher stable energy level, e.g., S_1_, without re-emitting energy. Instead of relaxing immediately to its original level (as in light scattering processes) a longer electron shift is caused. As a result, that incident wavelength is missing from the scattered light (Figure 3(A1C)). Many spectroscopic techniques use absorption spectra to identify compounds qualitatively or to measure concentrations quantitatively [127,158].

**Extinction as combination of scattering and absorption.** In some cases, techniques based on particle–light interaction techniques take advantage of both effects scattering and absorption [158]. Considering measurements of a particle suspension’s extinction *E* or transmittance *T* represent the sum of the of energy reduction caused by passing through the sample. Such effects are exemplary described by the extinction law, which illustrates the logarithmic ratio of the incident and the detected light intensity depending on the specific wavelengths. The combination of scattering and absorption may make a suspension of undissolved particles appear turbid and colored. The solution may appear either opaque (due to Mie scattering by larger particles) or opalescent (with areas of many colors due to Rayleigh scattering by small particles). The exact appearance depends on particle size and incident light wavelength, and varies with the diameter/wavelength ratio. Even though these are ensemble methods, they are sensitive and may allow useful estimates of the presence of particle as well as particle sizes.

#### 3.1.2. General Advantages and Challenges

The advantages of particle–light interaction methods include a wide variety that allows multiple investigation approaches and device configurations. They can be widely applied, without additional preparation or separation steps, to any suspension that transmits or scatters light—which includes almost all biopharmaceutical suspensions. The measurements are fast, nondestructive, and noninvasive. Additionally, they can directly or indirectly determine many parameters, including also non-optical properties such as diffusion coefficients or the interaction parameters A_2_ or k_D_.

The corresponding techniques are steadily improving, thanks to ongoing developments in camera, detector, and light-source (specifically laser) technologies.

However, there are also limitations. The processes, effects, and calculations are complex. The underlying Mie theory applies only to spherical particles. Multiple physical processes may be at work at the same time, possibly leading to false or distorted results. As published by Aleandri et al. (2018) one important parameter affecting the measurement quality is the DLS laser power. It was demonstrated that it is correlated to the resolution of DLS and therefore with the DLS detection limit [163]. Polydisperse particle mixtures with a broad size range are a challenge: high concentrations of small particles produce high background noise, or low concentrations of large particles can mask signals of more frequent smaller particles. Finally, resolutions are generally low, and detecting rare particle species may require separation-based techniques.

#### 3.1.3. Applications and Techniques

Particle–light interactions play a major role in particle detection and analysis. They include most compendial techniques, e.g., light obscuration (LO), nephelometry, and turbidimetry. LO is a single-particle detection method, listed in Ph.Eur. and USP, and used to quantify subvisible particles of 1–600 µm in diameter [25,26]. Particles pass a narrow light beam in a restricted volume (such as a flow cell). They cast shadows on a light detector, and the overall reduction in light intensity is proportional to the particle’s cross-sectional area. The analyst, however, must remain cautious, because transparent and highly translucent particles transmit some light, producing lower contrast shadows that can skew particle-diameter results (Figure 3(A2B)).

Turbidimetry and nephelometry are simple and highly sensitive techniques that detect light scattered at specific angles to the incident beam. Nephelometry measures intensity at a 90° scattering angle; turbidimetry detects transmitted light around a 0° scatter angle. The selection of the angles is based on the angle and size dependency of light scattering. For smaller particles (low nanometer range) the detection at an angle of 90° leads to the highest sensitivity as the signal-to-noise-ratio is best and inferences or diffraction are not destructive. For larger particles, in contrast, such destructive interferences damp the resulting scattering intensity at 90°. For this case the loss of scattering intensity, due to interference, is minimal at angles close to 0°. Neither technique provides detailed information on particle size or concentration, but they are highly sensitive in detecting the presence of particles.

Further examples of elastic light scattering techniques include static light scattering (SLS) methods: multiple angle light scattering (MALS) or right angle light scattering (RALS); laser diffraction (LD); dynamic light scattering (DLS). MALS and SLS devices measure scattering intensities at multiple angles to determine particle size and molecular weight. LD instruments detect specific diffraction patterns and based on these patterns the specific diffraction angles can give information about the particle size/shape. DLS uses scattered light to measure the particles’ Brownian motion to reveal their hydrodynamic diameter. By applying an electric field the electric mobility *µ* is determined by the scattered light to calculate the zeta potential of particles.

Typical examples for techniques detecting inelastic scattering or light absorbance are UV/Vis spectroscopy, IR spectroscopy, and Raman spectroscopy. These techniques are often applied in detectors of high performance liquid chromatography (HPLC) and AF4 instruments, such as UV or RI detectors.

### 3.2. Microscopy and Imaging

Microscopy is an extension of particle–light interactions, familiar to medical and biopharmaceutical research. In microscopic approaches, various physical principles and interactions are used to obtain a magnified image of the investigated objects. These applications have been described intensively and in detail elsewhere [164,165]. Here we will only give a short overview and concentrate on applications of particle detection.

#### 3.2.1. Principles

In the beginning microscopy was only realized by the usage of visible light microscopes. Modern visible-light microscopes can resolve objects down to 0.2 µm (about the *λ*/2 the Abbe limit for the shortest visible wavelengths), but further development has already increased resolution. The familiar optical microscopy instrument consists of two complex lens arrangements, the objective lenses and the ocular lenses (Figure 3(A2A)). Light passes from the source, interacts with the object, and is gathered by the objective lens. This real image is further magnified by the ocular lens into an image that can be detected by the eye or a camera system. The control of light refraction, in this case, enables the final imaging (i.e., reproduction of a real object based on specific parameters). To push resolution further down to 0.1–1 nm, it requires techniques like electron microscopy and probe scanning microscopy. Electron microscopes use an electron beam, with wavelengths in the 4-picometer range, to interact with objects. Probe scanning microscopes use a cantilever-mounted probe to detect changes in force or displacement as the probe tip moves over the object’s surface.

Based on these elements, imaging methods deliver detailed pictures of individual particles to develop information on particle size (or size-equivalent, based on cross-sectional area), a number-weighted size distribution, particle shape, transparency, and concentration. The obtained images are spatially resolved and color- or intensity-coded reproductions of the real objects. Particle sizes were originally determined by eye, from 2-D photographs. Today, the process is automated, using quantitative algorithms to score grayscale thresholds and measure pixel-based particle areas [101].

There are two basic modes of microscopic analysis: static and dynamic. In static analyses, such as TEM or scanning electron microscopy, particles are fixed to a membrane or filter, whereby the fixation itself may cause changes in sensitive samples. In dynamic analyses, such as micro-flow imaging (discussed in greater detail below), particles remain in suspension and flow past the imager. The basic measurement principle is the same in both types: particles enter a measurement zone, images of the sample are captured (by a CCD camera, for example), and the images are evaluated by analysis software. In dynamic analysis, the particles remain in their original suspensions, avoiding fixation changes, and the method can examine large volumes of sample in a given time. Such changes because of preparation, e.g., drying, is a popular challenge exemplary for TEM measurements. One potential approach to overcome such problems and to allow visualization of nanoparticles in solution is e.g., cryo-TEM [166].

#### 3.2.2. General Advantages and Challenges

The microscopic measurement methods, generally, enable the most direct detection and direct visualization of the sample. Images can be recorded to allow detailed evaluation or re-evaluation at any time. They allow observation of individual particles without separation (centrifugation, filtration, etc.). A direct particle quantification is realized yielding number-weighted particle size distribution data without estimations based on spherical models.

Microscopy, however, faces technical resolution limits, e.g., the Abbe resolution limit for optical microscopy. Sample preparations, such as sample isolation and sample fixing procedures, for some methods can alter particle properties. In general, PSD values are derived from small fractions of the sample, so they may not necessarily represent the entire sample. In most cases, values for particle size and concentration are derived from computer analyses, so the results depend on the selected software, algorithms, and threshold settings. Further challenges may arise from the detection principle and the sample properties. This is discussed in the following exemplary for optical microscopy: measurements based on light microscopy heavily depend on the wavelength of the incident light and the suspension’s optical properties (Figure 3(A2B)), such as the refractive indices of the particles and surrounding medium. Different combinations of these properties may make particles appear transparent, translucent, or opaque. The particles are transparent, if they have the same refractive index as the medium. Light is transmitted without refraction, scattering, or absorption, and these particles are difficult to detect. In contrast, a translucent particle’s refractive index is nearly the same as the medium’s. Some light obscuration and/or scatter makes imaging easier; as the difference in refractive index increases, so does contrast. It has been reported, however, that imaging techniques are more sensitive to translucent particles than, for example, pure light obscuration [167,168,169]. In the limit of highest contrast, the particles are opaque, transmitting no light at the most-absorbed frequencies and with small light-scattering effects (though diffraction is possible). Considering automation, static microscopic processes are difficult to automate, and high throughput is hard to achieve.

For flow imaging, on the other hand, automated, high-throughput systems are available that offer savings in time, cost, and labor.

#### 3.2.3. Applications and Techniques

There are many types of devices using the principle of microscopy and imaging, e.g., optical microscopes, fluorescence microscopes, FT-IR microscopes, transmission electron microscopes, scanning electron microscopes, or atomic force microscopes. Optical microscopy, however, is the technique prescribed by pharmacopoeias [140]. Microscopes are applied and essential in various fields and are continuously improved and developed. Prominent examples are medical approaches for cell and tissue studies or material investigations of structure and surface.

### 3.3. Resistive Pulse Sensing (RPS) or Coulter Principle

Resistive pulse sensing (RPS), also known as the Coulter principle or electrical sensing zone, was developed by Wallace H. Coulter in the late 1940s (and patented in 1953) for counting and sizing red blood cells [170]. The Coulter Counter enabled the first quantitative cell measurement based on volume. Today it remains the basis of many analytical methods and instruments—including a number of recent pore-based sensors. In the 1970s, De Blois and Bean published the first RPS methods for detecting submicron particles [171]. The principle was revised in 1996 with the first demonstrations of nanopore sequencing of single-stranded DNA [172].

#### 3.3.1. Principle

The principle and the configuration are simple, but powerful. A resistive pulse particle sizer device, or Coulter counter, consists of two compartments filled with an electrolyte solution with a conductivity, *ρ* (Figure 3(A3)). The two compartments, each containing an electrode, are connected through a small aperture or pore, with fixed length *L,* and diameter *D*. Applying a direct potential between the electrodes creates an ionic current flowing through the aperture. The resistance *R,* in the sensing zone around the aperture depends on the channel’s dimensions and can be described generally, by Maxwell theory (Equation (2)), or specifically for cylindrical channels as mentioned in (Equation (3)):

(2) $$\mathrm{Resistance}\;\mathrm{in}\;a\;\mathrm{general}\;\mathrm{pore}\;(\mathrm{Maxwell})\;\;\;\;\;R=\;\rho \int_{L}^{0}\frac{dz}{A\left(z\right)}$$

(3) $$\mathrm{Resistance}\;\mathrm{in}\;a\;\mathrm{cylindrical}\;\mathrm{pore}\;(\mathrm{de}\;\mathrm{Boise})\;\;\;\;\;R=\;\frac{4\rho L}{\pi D^{2}}$$

where the parameters are as follows:
*R*resistance*D*pore diameter*ρ*solution resistivity/conductivity*L*pore length*A(z)*cross sectional area perpendicular to z*z*length coordinate

Introducing a nonconducting (or weakly conducting) particle into the sensing zone around the pore reduces its cross-sectional area in the channel, proportionally increasing resistance and reducing the current. Conducting particles exhibit a strong electric double layer that disturbs the analysis and are hard to model in the mathematical approximations. For spherical particles, this resistance change, *ΔR*, is proportional to the volume of the particle. (Equation (4)):

(4) $$\Delta R=\;\frac{4\rho d^{3}}{\pi D^{4}}$$

where the parameters are as follows:
Δ*R*resistance change caused by particle passage*D*pore diameter*ρ*solution resistivity/conductivity*d*particle diameter

Current fluctuations can be recorded as voltage pulses (Figure 3(A3)). These data can be analyzed to determine total particle concentrations and individual particle size and shape. The pulse amplitude, *a*, is proportional to particle volume (or cubed diameter) (Equation (4)). The duration, *b*, and the shape of the impedance peak give information about the particle’s shape. The event frequency, *J*, (i.e., the number of peaks in the current graph), is proportional to particle concentration, *C*, as described by the Nernst–Planck relation (Equation (5)) [20]:

(5) $$\mathrm{Event}\;\mathrm{frequency}\;(\mathrm{Nernst}–\mathrm{Planck})\;\;\;\;\;\;J=\;\frac{\pi v_{S}D^{2}}{4}C$$

where the parameters are as follows:
*J*event frequency*D*pore diameter*ν_S_*particle velocity*C*particle concentration

#### 3.3.2. General Advantages and Challenges

The Coulter counter method is orthogonal to light obscuration and flow imaging techniques, and is independent of optical properties like refractive index or transparency. The method does not depend on optical contrast between particle and background, which might enable measurement of more high concentrated samples. In general, any particle that displaces liquid is detectable by RPS, without significant influence from particle composition or chemistry.

The RPS/Coulter principle allows particle-by-particle detection, capturing size and shape information for each particle. The measurement is fast, with good reproducibility and high resolution.

High-throughput measurements are possible where particle concentrations and flow rates are high. At low concentrations or flow rates, however, the method is time-consuming. RPS measurements can cover a broad range of particle sizes, but only by using multiple apertures. An individual RPS aperture can accommodate only a limited range of particle sizes. Signals from particles that are very small in relation to the aperture diameter get lost in the background noise. Particles that approach the aperture diameter can block the pore and generate nonlinear signals. Clearing these blockages takes time and effort, and they can alter the pore shape and size, affecting measurement results.

A well-formed cylindrical pore measures resistance in a small, well-defined region, yielding highly sensitive measurements. Calculations for pores with conical and other shapes, however, are more complex. For conical and other pore shapes the correlations become more complex.

For best sensitivity, RPS requires that particles are nonconducting, nonporous, and possess a narrow electric double layer. For samples in low conductive formulations (i.e., if the electrical resistance leads to electron motion impediment), electrolyte suspension can also become a critical factor as the current between the compartments might be insufficient for measurements. The researcher should also be alert to unspecific interactions between particles and the pore material, potentially leading to adhesion and finally to the formation of a particle layer on the pore material.

Since resistance calculations assume particles are spherical (Equation (4)), an RPS instrument must be calibrated with a particle-size standard to establish a correlation between a given resistance signal and a known particle diameter. In addition to the particle size, the pulse duration may give information about shape and the shape may also have an effect on the electrical current blocking behavior.

#### 3.3.3. Applications and Techniques

Several companies (such as Beckman and Symnex) followed Coulter-type technologies, and the development of the first flow cytometry instruments was based on RPS principle.

RPS instruments cover a wide range of applications, for food industries, paints, and toners to biopharmaceuticals and clinical medicine. The main application of Coulter-type devices is the quick and accurate analysis of blood (complete blood count CBC). There are a few examples confirming the application for the analysis of protein suspensions, containing, for example, BSA particle and IgG particles. Coulter-type counters also have potential, to fulfil the need for orthogonal techniques for the subvisible range, from a few nanometers to several micrometers.

Today, RPS apertures and benchtop instruments are expensive. The assays usually focus on the micrometer range, and require large sample volumes. There is a move to develop microfluidic Coulter counters on a chip, without fixed apertures. Several research teams have recently reported RPS devices that employ multiple apertures in parallel, hydrodynamic focusing, and adjustable or tunable apertures to attain smaller particle sizes, smaller sample volumes, and larger particle-size ranges [173,174].

### 3.4. Resonant Mass Measurement (RMM) and Suspended Microchannel Resonators (SMR)

Mechanical resonators have been used in chemical analysis for more than a decade to determine masses of organic and inorganic matter in gaseous environments. This period has seen significant progress toward the resonant mass measurement (RMM) devices directly measuring the masses of nanoscale particles and single molecules. These methods are highly sensitive, able to detect masses in the attogram range and even lower. Micro- and nanomechanical cantilevers are used widely where high sensitivity is needed, e.g., in atomic force microscopy [175,176,177].

#### 3.4.1. Principle

Existing mechanical resonator systems are most likely “dry” systems with a resonator situated in a vacuum environment and the investigated sample on top. Viscous damping makes it difficult to apply the method to measure particle masses in solution. Burg et al. (2003) developed a system of suspended microchannel resonators (SMRs) for biomolecular applications [175]. They described label-free biomolecular detectors able to measure cells in solution without significant viscous loss. The SMRs showed high quality factors (i.e., good resolution of small mass changes due to a light resonator with a pure tone) and extraordinary resolution, thanks to MEMS fabrication methods for constructing microelectromechanical systems (MEMS) that can precisely maintain a stable frequency.

These SMRs are microfluidic devices consisting of a tiny (≈100 µm with a mass of a few nanograms) mechanical cantilever-like resonator in which a microfluidic flow channel is embedded. Schematically the working portion of the device is shaped like a capital “T”. The top of the T is the fixed base, and the vertical line is the resonating beam. The resonator vibrates with a characteristic frequency *f* which is a function of its mass *m* and for SMRs also of the fluid density. The solution is pumped in at one side of the base, flows through a loop that goes out to the end of the resonator, and returns to exit through the opposite end of the base (Figure 3(A4A)). The resonant frequency drops as the beam’s mass increases, e.g., by a particle passing the tip of the cantilever, and this relation is applied to detect the mass of the particle (Equation (6)):

(6) $$f=\;\frac{1}{2\pi }\sqrt{\frac{k}{m^{*}+\;\alpha \Delta m}}$$

where the parameters are as follows:
*f*resonance frequencyΔ*m*mass change upon sample additionkspring constant of resonatorm*effective mass of the resonator*α*numerical constant depending on the geometric localization of Δm (as defined by [175,176])

The low mass of the beam makes the device very sensitive to the minute changes in mass as particles flow through the channel (Figure 3(A4B)). With careful design (e.g., isolating the resonator in a vacuum can yield data comparable to “dry” RMMs), SMRs can detect mass changes below the femtogram range.

Due to the microfluidic channel’s large ratio of surface area to volume, molecules and particles may adsorb to the channel walls; this can be exploited effectively for biomolecular mass sensing, especially if the interior surfaces are functionalized. Molecules can be directly detected as they bind to the walls inside the cantilever. Similar considerations apply to real-time cell growth experiments. In applications where molecules adsorb uniformly to the channel surface, the numerical constant α = 0.24 (Equation (6)) [176].

Another measurement mode applies to particle suspensions that flow through the resonator without significant interactions with the channel wall. The resonant frequency shifts according to the particles’ position and mass difference between the particle and the volume of medium it displaces (buoyant mass). The maximum shift occurs when the particle reaches the apex of the cantilever tip (Figure 3(A4C), position 2). At this point, α = 1 and the resonant frequency is proportional to the buoyant mass of the particle [176]. When particles enter the channel sufficiently separated far apart, frequency changes occur as well as separated peaks reveal individual particle properties. Particles less dense than the suspension medium produce “positive” peaks; denser particles produce “negative” peaks; particles with a density very close or equal to the medium’s are not detected. When peaks do appear, their amplitude (height) is directly proportional to the particle’s buoyant mass. The number of peaks is equal to the number of particles, so concentration is the number of peaks divided by the volume of fluid passing through the resonator. Frequency shift curves allow calculation of several particle properties:

Buoyant mass, *M_B_* and dry mass, *M*. As noted, *M_B_* is the difference between the particle mass and the mass of the fluid the particle displaces (Archimedes principle). The in vacuo or dry mass, *M,* is a function of the densities of the particle, *ρ_P_*, and the fluid, *ρ_F_* (Equation (7)):

(7) $$M=\;\frac{M_{B}}{1-\frac{\rho_{F}}{\rho_{P}}}$$

where the parameters are as follows:
*M*in vacuo or dry mass of the particle*ρ_F_*fluid density*M_B_*buoyant mass*ρ_P_*particle density

Subsequently, the equivalent spherical particle diameter, *D*, is based on the dry mass, *M*, (Equation (8)):

(8) $$D=\sqrt[{3}]{\frac{6M}{\pi \left(\rho_{P}-\rho_{F}\right)}}$$

where the parameters are as follows:
*D*equivalent spherical particle diameter*ρ_F_*fluid density*M*dry mass of the particle*ρ_P_*particle density

Particle density, *ρ_P_*: is obtained by measuring resonant frequency shifts for the same class of particles in carrier fluids with different densities [104].

Particle surface charge/zeta potential: Dextras et al. demonstrated the determination of surface charge/zeta potential based on the electrophoretic mobility [177].

Viscosity: Lee et al. described two different methods for determining viscosity, and demonstrated that SMR can measure viscosity with high precision [178].

#### 3.4.2. General Advantages and Challenges

In sum, RMM/SMR allows highly sensitive, highly accurate, particle-by-particle measurements with femtogram or lower resolution (comparable to, or better than, a high-end quartz crystal microbalance). The technique can simultaneously determine particle size, mass, density, surface potential, and concentration, along with the viscosity of the medium. The unique ability of RMM/SMR to differentiate between “positively” and “negatively” buoyant particles in the subvisible size range is important for pharmaceutical applications, for example to discriminate proteinaceous vs silicone oil particles [96]. Sample handling and preparation are easy: there is no labelling, no massive dilution, and no manual threshold setting.

Sample volumes depend on the device and the used microchannels, but are low as in the µL range, e.g., 100 µL for the Archimedes system (Malvern Instruments Ltd., Malvern, UK). The use of such low volumes is usually seen as an advantage. The current SMR are low sample throughput devices and to the best of our knowledge, no commercial “high-throughput” solution is neither currently available nor under development. The method depends on particle mass and density, so it is independent of optical properties. This means, however, that analysts must determine or estimate the densities of target particles, as well as understand and realize that particles with density closed to the surrounding medium may not be detected. The difference necessary to detect a particle depends on the sensitivity and accuracy of the device as well as the particle size (directly measured mass). For a polystyrene particle with a density of 1.05 g/mL (density difference to water of 0.05 g/mL) the particle should show at least a diameter of 220 nm [179]. Particles with a density difference of 0.03 g/mL (silicone oil in water), however, are detectable with diameters of 1 µm and larger [135]. Operators must be alert for shifts in sensor response, and periodic recalibration using standard particles (e.g., NIST traceable latex standards) may be necessary. Users must also remember that particle size results assume that particles are spherical; the data do not reveal the actual shape. Finally, it can be a challenge to avoid interactions between particles and the flow-channel walls, which can lead to clogging, changes in flow rate, channel blockages, and false results.

#### 3.4.3. Applications and Techniques

SMRs were developed to analyze biomolecules, and have been applied to a tremendous variety of questions. They have found special application in determining a cell’s physical properties, such as single-cell mass or density, to differentiate cells by type or growth phase. The ability to study particles in real time enabled investigators to follow cell growth [180,181,182]. Functionalized channel walls have been used to study molecular interactions, such as antibody–antigen interactions or avidin–biotin interactions [176]. Manufacturers must quantify and control drug loading of nanoparticles drug carrier systems. Nejadnik et al. showed how this can be done using SMR [183]. In another work, Son et al. suggested that SMRs might be just as useful as optical detectors in a wide range of applications such as HPLC, where SMR results proved to be as reliable as UV/Vis-detectors [184]. Patel et al. [96] also evaluated the SMR as general particle detection system for protein therapeutics. They stated that SMR can be used successfully to analyze both standard microparticles and subvisible particles in protein therapeutics. Such comparisons with established pharmaceutical techniques strongly suggest that SMR should be considered as a detection option in biopharmaceutical protein processes. In addition, the researchers suggested that SMR could be used for analyzing formulations of protein therapeutics without dilution, and to differentiate between particles of protein and silicon oil [96].

SMR also holds promise for studying protein-aggregation processes: Wang et al. gathered real-time data for characterizing the kinetics of proteins (human insulin) aggregating into amyloid fibrils [185]. The study demonstrated that the system enables to investigate changes in the environment of the investigated particles highly sensitive, which appears highly promising for various approaches.

RMM/SMR has been shown to be versatile in many applications [96,104,175,176,180,181,183,184,186]. Technical development continues, e.g., to achieve finer mass resolutions at the attogram scale to measure particles down to 10 nm with a throughput of more than 18,000 particles/hour, or to develop high-speed multiple-mode measurements to resolve particle position and mass [187,188]. So far, we know of only one commercial SMR/RMM system for analyzing micron and submicron particles, the Archimedes system (currently Malvern Instruments Ltd., Malvern, UK). The instrument covers a theoretical size range from 50 nm up to about 5 µm, depending on the sensor and the particle type. Practically the lower limit is set to 220 or 500 nm and up to about 3 µm [135,179]. SMR thus, has the potential to close the “1 µm-submicron gap”.

### 3.5. Separation Principles

Separation processes of heterogeneous systems are everyday processes that use a broad variety of underlying principles [189,190,191,192,193]. Separation of particle suspensions and biologicals, in particular, require understanding the particle’s properties as well as the roles of internal and external forces acting on them. Physically, particle suspensions are heterogeneous mixtures of solids and liquid. The solid phase itself is often heterogeneous, as well. This variation in particle properties is exploited to separate them—by differences in electrical charge, diffusivity, size, mass, density, or geometry. Separation is therefore often a strategy or approach for compensating the relatively narrow sensitivity ranges of many particle analyses and thus improving resolution in analyses of highly polydisperse samples. Performance will depend, however, on matching particle property differences, objective, and separation method. Once again, there is no one-size-fits-all solution.

The three main separation processes (Figure 3(B5)) are based on phase equilibrium, membrane exclusion, and external forces, respectively. Filtration, dialysis, tangential flow filtration, and diafiltration are typical membrane-based separation processes. Chromatography, extraction, and adsorption take advantage of phase equilibrium. External-force-driven separations, however, account for most of this category, via applications of centrifugal, electrical, magnetic, or thermal forces. Results of separation processes may be observed in real time (in some centrifugations, for example), or the fractions may be subjected to further analysis with detectors outlined in Section 3.1, Section 3.2, Section 3.3, Section 3.4, or by other means. A number of well-established approaches may combine these basic processes. These include sedimentation and centrifugation approaches, size exclusion chromatography (SEC), asymmetric flow field flow fractionation (AF4), and hydrodynamic focusing as used, e.g., in flow cytometry.

#### 3.5.1. Sedimentation and Centrifugation: Principle

Sedimentation is the common process in which suspended particles settle to the bottom of a container. As suspended particles move through a fluid, their behavior is influenced by three forces: buoyancy (*F_B_*), drag (*F_D_*), and gravity (*F_G_*) (Figure 3(B5)). If a particle is relatively large (i.e., not significantly influenced by Brownian motion) and denser than the surrounding fluid, gravity dominates and the particle sediments. The sedimentation velocity is determined by particle size, by the difference in density between particle and solvent, by temperature, and by interactions between the particles and their environment.

In general, the sedimentation behavior of spherical particles with known medium viscosity, *η*, and density, *ρ*, can be described by Stokes’ law:

(9) $$v_{S}=\;\frac{g\;\left(\rho_{P}-\;\rho_{S}\right)\;D_{P}^{2}}{18\eta }$$

where the parameters are as follows:
*v_S_*sedimentation velocity*ρ_S_*density of the solvent*g*acceleration due to gravity*D_P_*particle diameter*ρ_P_*density of the particle*η*viscosity

Stokes’ law applies only to low concentrations of non-interacting spherical particles with diameters significantly greater than 3–5 nm. Only these larger particles have sedimentation velocities great enough to significantly effect in time scales useful in the laboratory of manufacturing plant. Sedimentation rates drop as particle diameter decreases, and particles smaller than 0.1 µm have sedimentation velocities close to zero, as Brownian motion dominates the dynamics.

In centrifugal sedimentation, the centrifugal forces experienced by fast-spinning samples, overpowers gravitational force (Figure 3(B6A,B)). In this case, the sedimentation velocity of even smaller particles increases enough to overcome Brownian motion. This has, of course, made centrifugation a leading method for separating, detecting, and characterizing particles over a wide range of sizes, including those just a few nanometers in diameter. When centrifugal forces are considered, Stokes´ law is modified to:

(10) $$\ln\left(\frac{R_{f}}{R_{0}}\right)=\;\frac{\left(\rho_{P}-\;\rho_{S}\right)\omega^{2}tD_{p}^{2}}{18\eta }$$

(11) $$D_{P}=\;\sqrt{\frac{18\eta \ln\left(\frac{R_{f}}{R_{0}}\right)}{\left(\rho_{P}-\;\rho_{S}\right)\omega^{2}t}}$$

where the parameters are as follows:
*D_P_*particle diameter*ρ_S_*density of the solvent*η*viscosity*ρ_P_*density of the particle*R_f_*final radius of rotation*ω*rotational velocity*R*_0_initial radius of rotation*t*time to move from *R*_0_ to *R*_f_

The use of centrifugation processes has a long history (e.g., it has been documented in butter-making in the mid-15th Century) and Miescher used a centrifuge system for the separation of cell organelles in 1869. Svedberg developed the ultracentrifuge in the 1920s to generate the higher g-forces necessary for separating very small particles, like the serum proteins hemoglobin (about 5.5 nm in diameter). Today, we have a wide variety of centrifuges adapted for a wide range of applications, and their uses include studies of particle behavior and fractionation processes, as well as to separate particle populations into fractions for further analysis.

Laboratory centrifuges generally consist of a rotor to hold samples and an electric motor to spin them. The devices may be distinguished by speed regime (low-speed, high-speed, and ultracentrifuges) and rotor type. Analytical devices, in addition, may possess highly sensitive optical detection systems. In practice, there are two optical particle detection methods for centrifugal sedimentation: integral and differential detection (Figure 3(B6C)). In both methods, a detector beam passes through the sample at the same position along the axis of the sample tubes. In the *integral detection* method, a homogeneous suspension is loaded directly into the cell. At the beginning of the run, the particles are at their maximum concentration, and the transmitted light intensity is at its minimum. The particles settle in the cell, with the largest particles sedimenting out first. The concentration decreases and the transmitted light signal increases. The result is an integral representation that reflects cumulative dilution. From the centrifugal Stokes’ relation, the analyst can determine which particle diameters have settled out at each elapsed time; the signal intensity at that time is a function of the population of smaller-diameter particles that remain suspended. In the *differential detection* method, the sample suspension is placed on top of a clear liquid, and the transmitted light signal starts out at 100% of the supernatant’s light-transmission capacity. As particles and particle populations sediment, they obstruct the light beam and the signal decreases as a function of the diameter and concentration passing the beam at that moment. When all the particles have settled out, the transmission intensity returns to the initial maximum. Again, since the equivalent diameters of particles passing through the beam is a function of time, the plot of intensity over time reflects the particle size distribution.

#### 3.5.2. Centrifugation: Application and Techniques

Commercially available analytical centrifuges include for example the CPS Disc centrifuge (CPS Instruments Europe, NL) for differential centrifugation sedimentation (DCS) or the analytical ultracentrifuge (AUC) (by Beckman Coulter, Brea, CA, USA).

The disc centrifuge rotor is, as the name suggests, a hollow, optically clear disc. It uses the differential sedimentation detection method and the rotor is initially loaded with a fluid density gradient, commonly an 8–24% sucrose–water gradient. The sample particles are most likely suspended in a less-dense carrier, e.g., in this gradient water, and introduced to the cell at the “top” of the gradient. At centrifugal accelerations up to 2.4 × 10^4^ RPM, single-wavelength detectors can detect particles ranging from 5 nm to 75 µm in diameter. The AUC is optimized for accelerations up to 2 × 10^6^ g. The optical detection system uses mostly UV light absorption. The AUC is used for sedimentation velocity (SV), sedimentation–diffusion equilibrium (SE), or density gradient experiments. Biotechnological applications use SV more frequently than SE, because time plots of SV experiments can yield information on shape and molar mass, as well as size distribution. SE experiments analyze the final steady-state and can give information about (1) equilibrium constants, (2) molecular mass, and (3) protein–protein interactions.

AUC results are described by the Lamm equation. (Equation (12)):

(12) $$\frac{\partial c}{\partial t}=\;\frac{1}{r}\;\frac{\partial }{\partial r}\;\left(rD\frac{\partial c}{\partial r}-s\omega^{2}\;r^{2}\;c\right)$$

where the parameters are as follows:
*c*local concentration*s*sedimentation coefficient*t*time*ω*rotor angular velocity*r*radial position*D*diffusion coefficient

The literature includes many variations of methods and measurement approaches. One example is the differential centrifugation measurements, using a step-wise increase of centrifugation speed and/or time instead of a constant centrifugation speed. Particles are separated according to their masses and in particular for samples with a broad and heterogeneous particle mass distribution, e.g., cell crude, the particles are separated with better resolution. This technique is frequently used to separate cell organelles. Another example is density gradient centrifugations that fractionate particles according to their densities. Due to the density gradient particle samples are separated based on two essential parameters (acceleration and density) causing a slower separation and better resolution. In addition, **particles** of similar size, but different characteristics (e.g., nucleic acids and exosomes) are separated. Sucrose and cesium chloride solutions usually provide the supernatant gradient.

Sedimentation and centrifugation techniques can cover a broad range of particle sizes with good resolution, making them especially appropriate for highly polydisperse samples. While sedimentation and centrifugation themselves are independent of sample or device optical properties, the detectors needed to gather data depend on optical interactions. RI, MALS, UV/Vis, fluorescence detectors are commonly used. As described for other techniques before, analysts must also be aware that unstable particles may agglomerate or deagglomerate during centrifugation.

#### 3.5.3. Other Separation Principles

Other particle-separation methods used in biopharmaceutical sciences include various types of filtration processes, liquid chromatography (in particular size exclusion chromatography, SEC), and asymmetric flow field-flow fractionation (AF4).

In chromatographic methods, interactions (based on particle charge, hydrophobicity, diameter, or shape, for example) between stationary and mobile phases affect the rate at which particles in the mobile phase move through the column, so that different classes of particles have different retention times. 

*Size exclusion chromatography* (SEC): In SEC or gel permeation chromatography/gel filtration, particles are separated according to their hydrodynamic diameters relative to the pore size of the stationary phase (Figure 3(B7A)). Large particles, like protein aggregates, flow through the column, passing around the beads of the stationary medium; these particles do not penetrate the bead pores, and elute first. Smaller particles, like protein monomers or fragments, enter the narrow pores and increases retention time so that they elute later. SEC is mainly used either for SEC-based purification of molecules (e.g., antibody drug conjugates purification or distinguishing between different drug antibody ratio) or to monitor protein degradation by gauging the proportions of protein monomer, “soluble” (low-molecular weight) aggregates, and fragments. SEC is commonly used for monomer, dimer, and further low molecular weight particle; however, size ranges for individual separations depend on the pore size of the stationary medium. Particles too large for the medium can clog its resin pores. Therefore, in SEC a pre-filter/crude-catcher is used allowing just the analysis of small submicron particles.

On one hand SEC is robust, sensitive, and requires only low sample volumes. On the other hand, SEC cannot directly determine particle sizes. Separated fractions must be further analyzed by optical detectors, e.g., light scattering, UV, or RI detectors. The sample dilution and elution medium required by SEC can affect protein aggregate “stability” and conformation, particularly dilution alters the concentration of buffer salts. Sample dilution and its consequences may be the technique’s main drawback.

*Asymmetrical flow field-flow fractionation* (AF4): (AF4) is also based on the hydrodynamic diameter/behavior of particles. In contrast to SEC, though, AF4 has no stationary phase. The sample is injected in a horizontal flow channel, which consists of an impermeable upper wall, a spacer that defines channel height and shape, and a permeable membrane that forms the lower channel wall [194] (Figure 3(B7B)).

After the sample enters the channel, it is focused—consolidated into a small region by counter flowing current. Then a horizontal laminar flow carries the sample and medium over the lower permeable membrane. As they pass, an orthogonal crossflow pushes the particles into the low-flow-velocity region next to the membrane. The particles tend to diffuse back into the faster flow gradient of the central channel, but smaller particles diffuse more rapidly and emerge from the channel outlet first. Larger particles diffuse more slowly, accumulate at the low-velocity region of the laminar flow profile, and are eluted later—opposite to SEC.

AF4 works well with low sample volumes and provides a broad measurement range (particles from a few nanometers up to 100 µm in diameter). AF4 does offer lower resolution than SEC. Method-development may present challenges, but sample preparation is straightforward and the solution conditions are gentler and less prone to alter the sample. The user should be alert to possible interactions with the membrane material. Parameters like membrane material, molecular-weight cutoff, and the interplay between channel flow and cross-flow rates need to be chosen carefully.

Considering these factors, AF4 can be considered to complement both AUC and SEC, and in some cases (e.g., separation of submicron antibody particles) may be superior [104,107]. AF4 is well-established for analyzing nanoparticles and smaller protein aggregates, but methods for analyzing larger protein particles still require development [126,127,128]. These particles may demonstrate steric elution—in which retention is independent of the channel flow and applied crossflow rates, and depends only on particle diameter and the channel dimensions. In these cases, the particles elute directly after the focusing step is finished and can in most cases be evaluated only qualitatively.

## 4. Examples of Emerging and Alternative Technical Approaches for Biopharmaceuticals

### 4.1. Nanoparticle Tracking Analysis

Nanoparticle tracking analysis (NTA) is a recently commercially available method using microscopic imaging (usually dynamic) and particle–light interactions (i.e., dynamic light scattering), to determine size distributions and concentrations of particles in the nanometer diameter range, based on their Brownian motion. Since the first description of a commercial nanoparticle tracking system (Nanosight Ltd., Salisbury, UK) in 2006, NTA and PTA (particle tracking analysis) systems found applications in many industrial fields, and yet have become essential in biopharmaceuticals [93,118,119,120,121,122,123].

**Figure 4:**
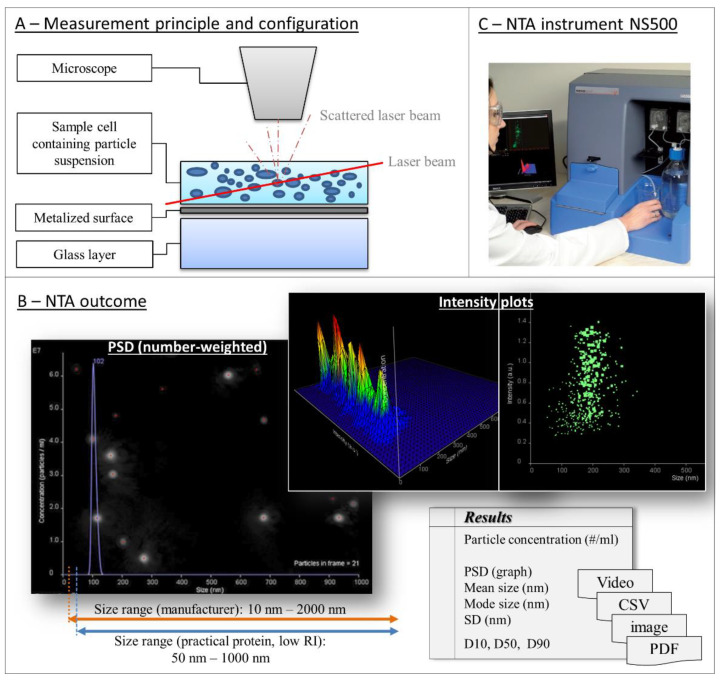
Nanoparticle tracking analysis (NTA): instrument, measuring principle, and final outcome. (**A**) Schematic illustration of the measurement principle of the NTA instrument (Nanosight Ltd.). The particle suspension is pumped into the sample cell where the laser beam encounters the particle causing light scattering. The light scattering centers of each particle are recorded and afterwards used to determine the Brownian motion of each particle indirectly by the motion of the scatter center. (**B**) Example of a measurement result using the provided instrument software. The recorded scattering centers are tracked and finally the number-weighted PSD, 2D and 3D intensity plots and a total particle concentration are provided. The results are obtained as video file, graph image files, text file (.csv), and PDF-file. (**C**) NTA instrument NS500 (Nanosight Ltd.). Adapted with permission from [195], Elsevier, 2011.

For a measurement the particle suspension is introduced into the sample cell (Figure 4A). As the particles jitter in Brownian motion, they are illuminated by a laser beam passing obliquely through the cell. A charge-coupled device (CCD) on a microscope above the sample tracks the particles’ light-scattering centers, creating a record of each particle in movement. Software analysis of the path data yields the diffusion coefficient, *D*, calculated via the Stokes–Einstein equation (Equation (13)) for each tracked particle. From this, knowing the temperature and viscosity of the suspension, we can calculate each particle’s hydrodynamic diameter, *d_H_*, the diameter of a sphere with the same *D*:

(13) $$D=\;\frac{\bar{{\left(x,y\right)}^{2}}}{4}=\;\frac{T\kappa_{B}}{3\pi \eta \;d_{H}}$$

where the parameters are as follows:
*D*diffusion coefficient*η*viscosity*T*absolute temperature*d_H_*spherical-equivalent hydrodynamic diameter*κ_B_*Boltzmann´s constant



As each scattering center is tracked separately, the resulting estimate of particle size distribution is a direct count, and not an intensity-weighted, z-average distribution (a common limitation of conventional ensemble methods, such as DLS) [124,125,126,127]. NTA results can be presented as concentration-based PSD histograms, or as intensity plots (Figure 4B). Intensity plots help to estimate the polydispersity or even to differentiate between particle with high RI and particle with low RI [128,129]. Calculations can reveal total particle concentrations in the range of 10^7^–10^9^ particle/mL, mean and mode size, as well as the parameters D10, D50, and D90, which describe the PSD in more detail.

A few comparative studies highlight where NTA has potential advantages over established methods such as dynamic light scattering, resonant mass measurement, and differential centrifugal sedimentation [120,130,131,132]. In 2010, Filipe et al. published the first critical evaluation of NTA in a biopharmaceutical application, focusing on the analysis of biologics [196]. The authors concluded that NTA does offer direct, real-time visualization of particle movement, while simultaneously counting and sizing particles ranging from approx. 30 nm to 1 µm (with some variations in size range limit, depending on the particles’ properties). For protein samples they set the lower size limit to about 50 nm. According to Filipe et al., NTA provides slightly better resolution than DLS [126]. Another study by Anderson et al. did not find the NTA resolution superior for the tested samples in comparison with other techniques [131]. Elsewhere, van der Pol et al. have recently shown that differences in refractive index of different particle populations may help to differentiate single nanoparticle populations by both size and scattering intensity, with the scattering intensity providing information about shape and composition [85,197]. These additional read-outs make NTA a preferred tool for analyzing polydisperse samples. By the same token, however, the flexibility and variability in parameter settings make the final results operator-dependent and thus objective results are questionable as recently shown [145]. At the same time, methods that depend strongly on operator skills can produce results that vary from user to user, making comparability uncertain. As a practical matter, particles with low refractive indices (RI)—like protein particles (RI ca. 1.34–1.4, l = 589 nm at 20 °C) in water (RI of water ca. 1.334, l = 589 nm at 20 °C)—scatter light only weakly, which makes analysis a challenge [145]. Nonspherical particles with diameters above 500 nm show oscillating scattering centers in NTA measurements. Additionally, highly concentrated suspensions, with 10^7^–10^9^ particles/mL, usually require dilutions can change the sample composition. The technique of NTA was shown to be a versatile tool, also for biopharmaceutics, but the dependency on the operator parameters may lead to low comparability, i.e., high user-to-user variability.

### 4.2. Micro-Flow Imaging

Micro-flow imaging (MFI), described by Sharma et al., is an increasingly popular method that uses dynamic microscopic imaging to determine particle size distributions, concentrations, and shape properties [101,168]. The first commercial MFI instrument was presented by Brightwell Technologies in 2011 [101,198]. Today, several manufacturers offer devices based on MFI or closely related principles, and MFI has emerged as one of the techniques with the greatest potential in biopharmaceutical subvisible particle detection.

**Figure 5:**
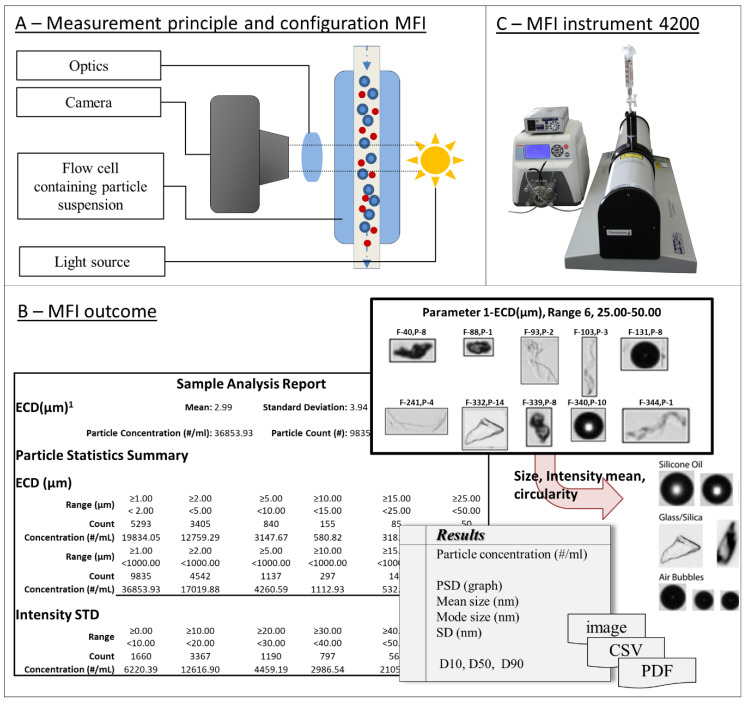
Micro-flow imaging (MFI): instrument, measuring principle, and final result. (**A**) Schematic illustration of the measurement principle of the MFI instruments (Protein Simple). The particle suspension is pumped into the flow cell where the camera captures bright field images as raw data. (**B**) After analyzing the images by an imaging software considering the amount and the greyscale of each pixel a report is generated. This report contents number-weighted PSD as well as example images of single particles. Due to properties like size, intensity mean, or circularity, filters can be applied to obtain and analyze particles with specific properties. The results are obtained as particle image files, text file (.csv), and PDF-file. (**C**) MFI instrument DPA 4200 (Protein Simple).

In MFI, a simple fluidic system passes the sample through a flow cell (Figure 5A), where it is illuminated by a pulse of bright light. Particle motions/images are recorded using a custom magnification system with well-defined magnification and an extended depth of field. For increased sensitivity, the instrument performs an automated calibration before each sample run, using low-noise electronics to optimize detection thresholds. Additionally, as the motion frames are captured, software algorithms are applied to further reduce noise and compensate for variations in spatial and pulse-to-pulse illumination. Image analysis software identifies each particle, tracks it frame to frame, and extracts and records information on size, shape, and contrast. Raw images are saved as PDF files, and particle parameters are saved to a database, and can be exported as a spreadsheet. Post-experiment software analyses of the particle database produce information on counts, concentrations, and characteristic distributions (such as number-weighted PSDs). Image filtering (e.g., for silicon oil) can identify and isolate subpopulations based on particle shapes and grayscale image values. (Figure 5B) The results can be presented as concentration-based PSD showing size distribution, corresponding and particle concentrations—reported, for example, in the particle diameter regions (<10, <25, and >50 µm) generally of interest to regulatory authorities [89,168,198,199].

Numerous studies have applied MFI, e.g., in cytometry or for biopharmaceutical particle detection [200,201,202]. Huang et al. compared LO and MFI accuracy in sizing and counting subvisible particulates [100]. Results showed the trends of both methods in high agreement, but concluded that MFI was more sensitive in the analysis of translucent particles, such as proteins and detected about 100 times higher particle concentrations [100]. Sharma et al. further demonstrated the applicability of MFI for protein samples [101,168]. MFI’s detailed visualization (compared to LO or dynamic light scattering) can be applied to studies of induced aggregation, and to differentiate between protein and other process particles. In polydisperse samples, for example, MFI can, without preparative separation distinguish between protein and silicone oil particles in the range above 10 μm [135]. MFI software allows sorting and grouping particles by specified shape and reflected signal intensity values, and nowadays, MFI systems have been automated for high throughput using an auto-sample system, such that in a single run up to 96 samples without manual intervention are handled.

There are also drawbacks. Developing the analytical algorithms is challenging, and the advanced analytical software can produce skewed results, if the operator chooses the wrong parameter settings (such as an inappropriate gray-scale threshold). As a particle-by-particle approach, flow imaging microscopy gives detailed results on a relatively small portion of the overall sample, and does not necessarily reflect the properties of the sample as a whole (though careful attention to sampling statistics in specifying appropriate volumes can generate representative results). The instrument may have limits on the number of particles it can detect, count, and file parameters in the database, so suspensions with high particle concentrations might require dilution—which, as noted, can alter key sample properties. Though MFI can discriminate smaller refractive-index differences between particles and media better than other techniques can, there is still a limit below which particles become nearly transparent and undetectable [35]. This can be a particular issue in suspensions that combine high protein concentrations with buffers containing a number of excipients [169,203].

MFI’s detection range (particles (2), 5 to 100 μm in diameter) is similar to that of light obscuration, but MFI yields more information on shape and material and does a better job characterizing translucent particles. This makes MFI very promising also for regulatory applications [25,26,27].

### 4.3. Tunable Resistive Pulse Sensing (TRPS)

*Tunable resistive pulse sensing* (TRPS) was developed to address shortcomings in the original Coulter resistive pulse sensing technique. In the literature, TRPS is also called *scanning ion occlusion spectroscopy* (SIOS), *size tunable pore sensing* or *tunable elastomeric pore sensing* [143,204,205]. TRPS was first described and patented by Sowerby et al. in 2007 [206]. Almost three years later, Willmott et al. presented the first commercial nanopore platform, a Coulter-type instrument employing TRPS to determine particle size, concentration, and charge [207,208]. Today, at least one manufacturer (Izon Science, Ltd. Oxford, UK) produces TRPS-based instruments for nano- and micro- scale particle analysis.

**Figure 6:**
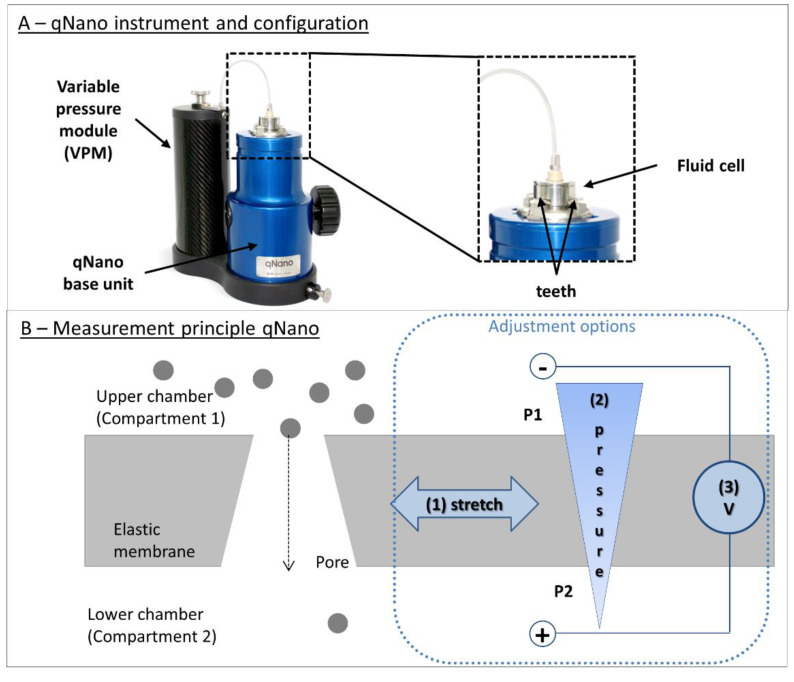
Tunable resistive pulse sensing technology (TRPS): instrument, specific pores, and measurement principle. (**A**) Configuration of the qNano instrument using the TRPS technology (Izon Science Ltd.) zooming from the complete device (left) to the specimen with the tunable nanopore (right). Image adapted with permission from a product of Izon Science Ltd., 2020. (**B**) Schematic illustration of the measurement principle of the qNano instrument. The suspended particles in the upper compartment pass through the pore and cause the resistive pulse. There are three options to adjust the instrument for optimal settings for the specific sample: mechanical pore stretching, pressure difference adjustment, and the applied direct current voltage (right).

The measurement principle is a variant of RPS (Coulter principle). TRPS replaces the fixed-diameter pores of RPS with dynamically resizable nanopores for more flexible real-time particle detection, quantitation, and characterization [209,210]. The system consists of a base unit, the so called (qNano), a fluid cell, and a variable-pressure module (VPM) (Figure 6A, left). The heart of the device is the pore, which is carried on a septum mounted in a cruciform specimen (Figure 6A, right). Tungsten needles of precisely controlled diameters create the pore by punching through a 200-µm thick elastic polyurethane membrane [211,212]. By varying the needle dimensions and the puncturing process, the manufacturer can produce pores of varying geometries in diameters that range (currently) from 50 nm to 10 µm [213]. Like all Coulter-type counters, the TRPS system includes a fluid cell consisting of two compartments connected through the pore. (Figure 6B) Sample is introduced into the upper chamber, and current is applied across the membrane. As in conventional RPS, a particle passing through the pore changes the resistance and produces a pulse in the current. Additionally, also as in conventional RPS, this pulse record can be interpreted to give information on particle diameters, counts, size distributions, concentrations, shape, and charge. Unlike its predecessor, however, the TRPS system offers three additional controls (Figure 6B, right) [209,214]:

(1) Operators can change the pore’s shape. The pore specimen is mounted between inlet and outlet fluid chambers with the limbs of the cross gripped in teeth that can move inward and outward in the plane of the specimen. This movement stretches the pore, changing its diameter and shape to suit the particles being analyzed. This is finally checked by the baseline and an appropriate standard.

(2) Operators can change the pressure gradient between the two chambers via the variable-pressure module (VPM). This adjusts the fluid flow through the pore independent of the particle charge. An optimal fluid flow is essential for obtaining the right pulse frequency and duration (also see below—optimal assay set-up). The VPM allows the measurement of samples with low particle concentrations down to 10^5^ and up to 10^11^ particles/mL; reverse flow is also possible.

(3) Operators can adjust the voltage. This helps optimize the signal-to-noise ratio and can even induce electrophoretic movement. In particular, the last two mentioned variables need to be controlled and balanced for the optimal assay set-up. Optimal set-up means that each particle that is detected should give a separate “peak” that can be differentiated from the background noise. The optimal voltage guarantees sufficient current for the baseline, i.e., for a stable baseline signal. To separate the peaks the particle velocity through the pore is adjusted by the applied pressure and/or by electrophoretic motion.

**Figure 7:**
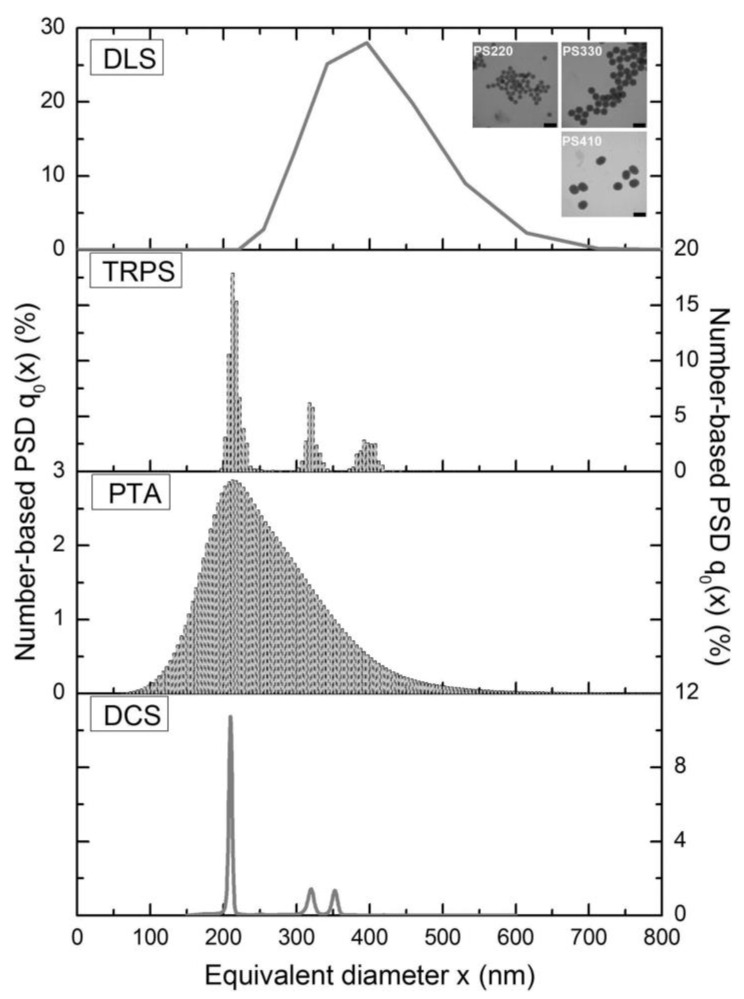
Comparison study of dynamic light scattering, tunable resistive pulse sensing, nanoparticle tracking analysis, and disc centrifugation concerning the resolution of a trimodal polystyrene particle suspension. Three polystyrene particle standards with sizes of 220, 330, and 410 nm—as seen in the images—have been analyzed as a trimodal mixture. This mixture was subsequently analyzed by dynamic light scattering (DLS), (nano-)particle tracking analysis (PTA), disc centrifugation (DCS), and tunable resistive pulse sensing (TRPS). In result only TRPS showed sufficient accuracy, resolution, and precision for the polymodal suspension. Adapted with permission from [34], Elsevier, 2013.

TRPS has been included in several comparative evaluations of established and emerging particle-analysis techniques [34,209,210,211,214,215]. Anderson et al. compared DLS, PTA/NTA, DCS, and TRPS [34]. They assessed populations of polystyrene (PS) spheres with diameters of 220, 330, and 410 nm, first in separate populations and then in multimodal mixtures. All four of the tested techniques could accurately assess same-diameter populations in all three size ranges as long as homogeneous, monomodal samples were investigated. In multimodal mixtures, however, only TRPS could clearly resolve each of the three particle-size populations as a discrete peak (Figure 7) [34]. In a study on the effect of particle concentration, van der Pol et al. found that TRPS measurements matched the TEM reference values better than did flow cytometry or NTA [216].

TRPS may thus be considered a highly accurate and precise method that outperforms other particle analysis techniques in resolving polydisperse or multimodal populations. The technology addresses RPS’s principal drawbacks, pore blockage, and limited response range for polydisperse suspensions. Increasing pore dimensions increase the upper limit of particle diameters TRPS can handle, of course. Decreasing pore dimensions increase signal to noise ratios for particles at the lower edge of resolvability. Moreover, the ability to simultaneously determine nanoparticle size, concentration, and charge is unique among the methods we reviewed here.

TRPS pores are not immune to clogging, however. Particles that are larger than the pore diameter, or have a high affinity for the pore material, can still block the channel. Membrane coating agents improve data quality by reducing adhesion and chemical reactions between the polyurethane and components of the suspension, especially in biological samples [213]. Moreover, pore elasticity and the ability to reverse the fluid flow make it easier to recover from pore obstructions without interrupting an experimental run.

Despite their wider detection range, it can be challenging to analyze very highly heterogeneous and polydisperse samples. Adding a preceding separation step, such as an SEC column, can help [217]. Measuring zeta potentials is possible but can be difficult, depending on the material. The mathematical model used to interpret instrument readings may not fully account for the effects of pore geometry, and they fluctuate a bit when particle and pore diameters are nearly the same. Calibration with standard particles is necessary to ensure TRPS comparability from one run to another. This assay protocol includes the calibration with particles of known size to ensure the correct comparability. The whole system is under further development for new applications and approaches. To the best of our knowledge no study has been published so far showing the successful application of the TRPS for biopharmaceuticals, in particular monoclonal antibody suspensions.

### 4.4. Flow Cytometry

Today, flow cytometers are the workhorses of cell biology research, medical diagnostics, immunology, and hematopathology. Flow cytometry pioneered the automated, qualitative, and quantitative, multiparameter analysis of cells in suspension. Measurable parameters—such as cell size, count, and morphology—are also of interest for particle detection and characterization [218]. Flow cytometers evolved from the Coulter counter (q.v.) systems for blood cell counting around 1953 (by W. H. Coulter), and were further optimized when Crosland-Taylor used laminar coaxial flux properties to align cells in a capillary by hydrodynamic focusing [219]. The first automated fluorescence-based flow cytometers appeared in the 1960s. In 1969, van Dilla described the forerunner of today’s devices, a non-microscopic instrument that used fluorescent dyes and an integral laser-based fluorescence-detection system [220].

**Figure 8:**
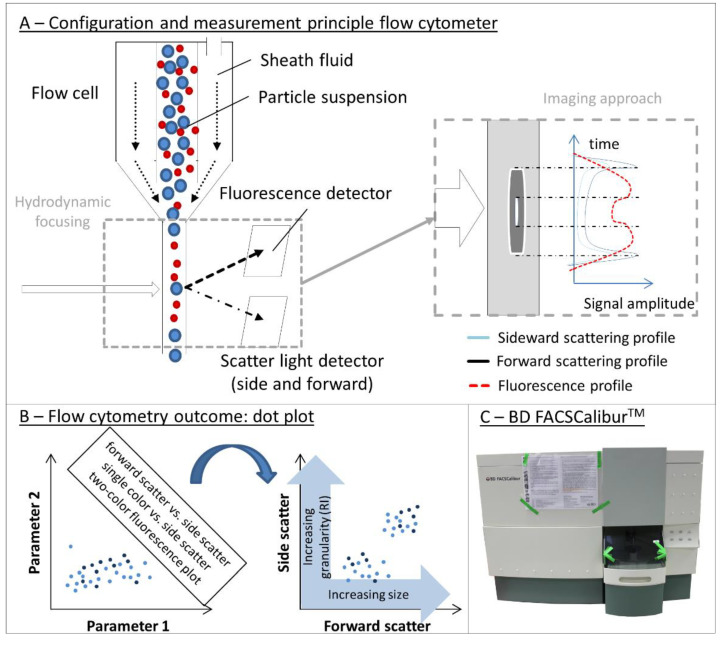
Flow cytometry (FC): instrument, measuring principle, and final outcome. (**A**) Schematic illustration of the measurement principle of a flow cytometry instrument. The particle suspension is injected into a flow cell. On the outer side a sheath fluid is applied and results into a hydrodynamic focusing. After this focusing the suspension enters a narrow capillary. At a fixed position of the capillary a light beam encounters the particle and the fluorescence signal as well as the scattered light (sideward and forward) is detected (left part). An emerging approach is the recording of a specific signal profile (right part) which leads to a more detailed characterization and even visualization of each particle. (**B**) The final results of FC measurements are dot plots considering multiple detected parameters, e.g., fluorescence, forward scattering or sideward scattering. (**C**) Exemplary, the BD FACS Calibur as a flow cytometer enabling particle detection and characterization.

The flow cytometry technique combines several concepts. The main features of flow cytometers are hydrodynamic focusing and laser-activated particle–light interactions (fluorescence and/or scattering) (Figure 8A). Some instruments are also able to modulate an electric field to sort droplets (particles) by diverting them into one of two or more flow paths. Flow cytometers combine three components: a fluidic liquid system, an optic system, and an electronic system [218]. The liquid system transports the particle and focuses the particle stream at the measuring point. Entering particles are injected into the axis of a cylindrical laminar-flow sheath. The particle-and-sheath stream is funneled and accelerated into a narrow linear particle flow (this is the hydrodynamic focusing increasingly used in microfluidics). The result is a single line of well-spaced particles that passes down the capillary and through the laser beam.

The optic system comprises the stimulating light sources and the detectors. Laser beams of different wavelengths are combined and passed through lenses that form and focus the beam. The laser light is scattered by the particles in the sample stream and it excites characteristic fluorescence signals of cell components or exogenous markers. The scattered and/or emitted light is collected, and the signal is directed through beam splitters and filters to separate the signal into its component wavelengths. The optical signal is finally detected by a photomultiplier or photon detector.

The electronic component transforms the optical signal into an electrical pulse, which is amplified and converted into a digital signal (Figure 8B). The dot plot, an example of the result output, displays each particle’s detector readings as a function/correlation/dependency of two parameters (such as fluorescence and scattering) on an x-y plane. Particle concentrations are directly determined via the particle or cell count; other properties may be more complex and require more analysis. Particles in the sample stream may or may not have intrinsic fluorescence. They may be stained or unstained. Even if the particulates are unlabeled and lack inherent fluorescence, they do produce measurable scattering, both forward scattering (FSC) at small angles and side scattering (SSC) at 90°, that can be interpreted via Mie theory. FSC intensity can be correlated to the relative size of the passing particle and SSC as a result of diffusion, reflection, and refraction processes about the structural complexity as well as physical properties of the particle (Mie theory) [218]. As in conventional light microscopy, carefully selected fluorescent dyes and markers can reveal substantial additional information about a cell or particle.

The use of flow cytometry as particle detection and characterization method has been demonstrated in previous studies [216,221,222,223,224,225,226]. Mach et al. applied flow cytometry for the determination of subvisible particle concentration in antibody formulations for particles larger than 1 µm [222]. Filipe et al. stated that fluorescence-labeled subvisible IgG aggregates can be detected down to 200 nm of size [223]. The use of specific fluorescence dyes, like the hydrophobic dye SYPRO orange or BODIPY for silicon oil staining, further helps to characterize the particles [221,222]. The addition of such fluorescence dyes and the resulting interactions with the sample, however, might cause unexpected or undesired effects, such as structural changes, and from the practical point staining procedures are less desirable. In 2014, Nishi et al. applied flow cytometry for the label-free detection, quantification, and characterization of unstained subvisible antibody particles down to 500 nm [224]. In addition, particle characterization based on physical properties, such as density or morphology was performed [224]. Furthermore, the authors evaluated the influence of parameters, such as variable flow rates, and showed proportional correlations between flow cytometry, MFI, and LO results.

Overall, the hallmarks of flow cytometry are speed, high throughput, sensitivity, the ability to measure many parameters simultaneously, and the capacity to physically sort cells [218,226]. The determination of size is most likely realized by clustering the detected events based on particle properties of calibration standards. For that reason, no direct size determination is possible. Flow cytometry lends itself to high-throughput screens, using multiwell plate systems, over a theoretical particle size range of 0.1 to 100 µm, which covers the subvisible particle regime. Measuring FSC and SSC together can produce read rates of up to 5000 particle/s, and accommodate sample volumes as small as 100–200 µL [222,223,224]. The identification and variability of parameter settings for measuring and evaluation may indeed appear challenging [224].

Current trends in flow cytometry include single-cell spectroscopy and mass spectrometry, and imaging individual cells in motion or even adherent [218]. Several manufacturers have combined flow cytometry and imaging, e.g., the ImageStreamX or FlowSight instruments (Amnis Corporation, USA/EMD Millipore Corporation, Darmstadt, Germany). The CytoSense instrument, (CytoBuoy b.v., Woerden, The Netherlands) combines the classical data collected by flow cytometry to build in silico images of the particles [218]. Based on the detected fluorescence and scatter profiles a pulse shape of the particle can be obtained, which allows the monitoring of particle features and internal structure (Figure 8A) [58,227]. Such hybrid systems are notable for their ability to gather individual flow cytometric data, multiple data points per particle, and particle images. Further developments of optical systems will raise expectations to enhance in precision and detection limits as well as novel application fields, such as particle detection in biopharmaceutical research.

### 4.5. Space- and Time-Resolved Extinction Profile (STEP^®^-Technology)

The space- and time-resolved extinction profile (STEP) technology was developed by L.U.M. GmbH (Berlin, Germany) and first commercialized in 1998 for use on sedimentation processes. In 2004, the same manufacturer released an optimized centrifuge instrument (LUMiSizer^®^) for characterizing nanoparticles and analyzing their dispersion stabilities. Instruments using the STEP^®^-Technology are still evolving, with the addition of different light sources and suitable detectors for the wavelengths 470 or 865 nm enabling multi-wavelength (2012) and X-ray (2015) devices.

**Figure 9:**
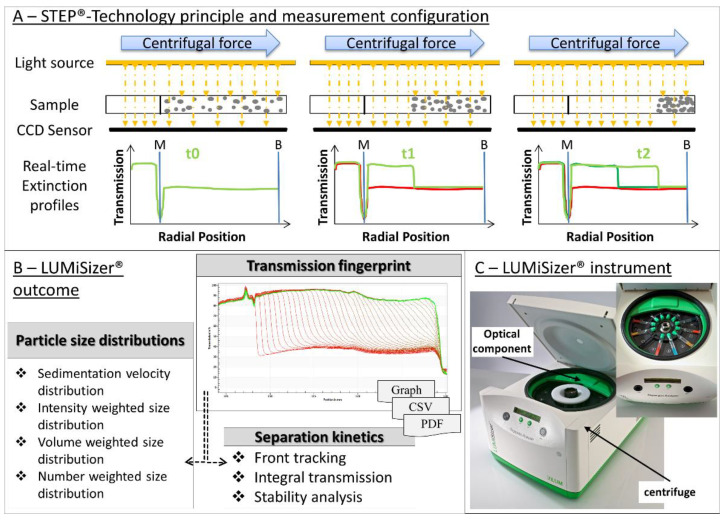
Space- and time-resolved extinction profile (STEP): instrument, measuring principle, and final outcome. (**A**) Schematic illustration of the STEP^®^ technology measurement principle. The particle suspension is homogenously distributed. Light is applied the over the whole sample and the transmission light is detected behind the sample over the whole lengths. The application of a centrifugal force leads to particle movement from the meniscus M to the cuvette bottom B. The clarification over time and a specific sedimentation velocity can be calculated. (**B**) The direct result achieved is the transmission fingerprint. Based on this various data analysis possibilities are provided by the software. Depending on the information known about the sample particles a wide variety of analysis and data evaluations are possible. The results are obtained as video file, graph image files, text file (.csv), and PDF-file. (**C**) LUMiSizer STEP^®^ instrument (LUM GmbH) Image adapted with permission from a product of LUM GmbH, 2020.

The STEP^®^-Technology instrument, an “analytical photocentrifuge” extension of the analytical ultracentrifuge with lower centrifugation speeds of maximum 2300 g, combines sedimentation separation with real-time optical transmission/absorbance measurements (Figure 9). The specific feature of the LUMiSizer^®^ is the optical setup for detecting real-time sedimentation. Sample cuvettes, with minimal volumes of 0.4 or 1 mL, are loaded into slots of a centrifuge rotor. As the sample spins (6–2300 g), it passes through the light—multiple beams that pass through the sample at many defined points along the cuvette’s long axis. Each beam is paired with a CCD sensor. The result is a record of light transmission and absorbance at each height in the cuvette at fixed time intervals over the length of the run (Figure 9A). The incident light wavelength can be chosen to suit the sample: near-infrared, blue light for highly transparent samples, or X-rays for highly concentrated, extremely turbid samples and for gradients within sediments.

In operation, the STEP^®^ instrument functions like “multiple device”, combining the fundamentals of sedimentation with a specialized optical detection system for transmission/absorbance measurements. This multi-point data-gathering capacity provides the eponymous space- and time-resolved extinction profiles. The device accelerates particles up to 2300 g and captures extinction coefficient information for every rotation at each level of the cuvette, as described by the Lambert–Beer law.

STEP^®^ produces a set of extinction profiles, a transmission fingerprint, and the software offers a number of options for analyzing the data (Figure 9B): sedimentation velocity, particle size distribution (from Stokes, Equation (9)), using a simple correlation (Equation (14)):

(14) $$u=\;\frac{r_{m}ln\frac{r_{m}}{r_{0}}}{t_{m}}$$

where the parameters are as follows:
*u*sedimentation velocity*t_m_*measurement time*r_m_*measurement position*r_0_*meniscus position

Centrifugation accommodates a wider range of particle sizes (down to 10 nm and up to 100 µm) than other orthogonal principles, and is correspondingly widely used. This type of analytical centrifugation (AC) has, naturally, advantages and disadvantages when compared to the two most used centrifugation techniques, AUC and DISC. The main difference between AC and AUC is rotor speed. AUC uses higher, fixed rotor speeds up to generate higher accelerations and better resolutions, as low as a few nanometers in a reasonable time of 6–12 h. Consequently, AUC is widely considered the most accurate technique. On the other hand, AUC requires excellent operator skills and substantial experimental effort: working with the complex cuvettes/sample cells in the vacuum needed to attain these high speeds and accelerations demands time and care. AUC’s fixed rotor speeds limit the technique’s dynamic range, and the very high accelerations may damage unstable samples (by contributing to agglomeration and aggregation, for example). Thus, the variable lower rotor speeds of DISC or AC might be beneficial where dynamic range and sample stability are concerns. The major drawbacks of DISC are that a calibration concerning the density zones is necessary and that the density gradient might alter the sample properties.

AC using STEP^®^ technology is highly suitable where the sample particles’ mass and density allow sufficient sedimentation already at low rotor speeds. Furthermore, there is no calibration necessary; temperatures can be controlled; samples, instrument, and software are easy to handle; very turbid samples can be successfully analyzed; up to 12 samples can by analyzed in a single run. Fast runs are performed for about 2 h, but depending on sample properties measurement times may be up to several days. As Walter et al. demonstrated, AC with STEP results for particle size and particle size distributions are in excellent agreement with AUC [228,229]. The determination of further particle properties, e.g., density or shape, is another approach supporting the analysis and enables the application in a wider field. However, centrifugation as an ensemble method is no particle-by-particle technique and quantitative evaluations are hard to achieve. In addition, the required material data, like particle density, are hard to determine in particular for aggregated protein samples. Another remaining challenge is the analysis of high concentration samples, e.g., HCLFs with up to 200 mg/mL or particle suspensions up to 90 vol%. If such concentrations of particles are present, hindered settling and multiple scattering occurs. These phenomena can be considered in new hindering functions for evaluation and improved analysis approaches as shown by Walter et al. [228,230].

Since the release of the instruments, several studies are published to show AC is a versatile tool for PDS, stability analysis, separation velocity, interactions, or structural stability/rheology in various fields: cosmetics, food processing, and material science, as well as biopharmaceuticals. Bharti et al. applied STEP^®^ technology to investigate interactions among nanoparticles as part of an effort of engineering functional structures depending on the used formulation conditions [231]. Further verification as an applicable technique in pharmaceutical development was shown for the characterization of vaccines, approaches using nanoparticles and microgels and even for formulation development of various compounds. To date, STEP^®^ technology does not appear to have been used to develop formulations of proteins (such as therapeutic antibodies) on the nanoparticle scale. Experiments are underway to study these applications.

## 5. Conclusions and Outlook

Though particle detection and analysis are of tremendous interest in research and industry, there are no universally applicable techniques or measurement principles suited for all types of particles because of tremendous differences in particle properties. Especially, biopharmaceutical formulations display numerous challenges that, until now, cannot be overcome by one single technique. They do not only cover a broad size range from few nanometers to several tens of micrometers but also present heterogeneous and polydisperse optical and material properties (e.g., refractive index, particle density, multiple degradation and/or aggregation pathways, highly dynamic confirmation, highly sensitive to changes in the environment). For that reason, researchers must carefully select methods from a wide range of available options, matching the method to the application and the specific characteristics of the sample. Failure to duly consider the analytical technique’s idiosyncrasies can lead users to over-interpret or misinterpret results.

This article presents the current most-evaluated principles of particle analysis and detection, and discusses the tremendous variety of specific techniques that embody them, their effects on particle interactions, the data that could be obtained, the knowledge that could be gained, and their advantages and disadvantages (Table 1).

Long-established principles, such as particle–light interactions, still produce state of the art methods and instruments. Although drawbacks in regard to biopharmaceuticals (e.g., the assumption of spherical particles, the high polydispersity of the particle mixture, and the complexity of the underlying theory) hamper the broad applicability, properly analyzed particle–light interaction studies yield substantial information about particles and their properties, especially size and size distribution. Therefore, the majority of techniques and detectors are based on this principle. If operators were fully aware of the complex calculations, such as Mie theory approximations, and the impact of various effects, misleading interpretation of the data and biased results could be avoided.

In this context, orthogonal principles become highly important. While centrifugation, for example, is mainly used for preparative purposes, analytical centrifuges offer great potential in detecting and analyzing particle populations that display a broad size range and are highly polydisperse. However, for centrifugation based methods, sample throughput is often very low. Common particle-by-particle approaches—Coulter counters/RPS and flow cytometry generally—offer close up views of individual particles that can provide essential information on bulk behavior. Nevertheless, there are certain challenges in terms of biopharmaceuticals that the operator has to be aware of. RPS instruments usually require large sample volumes and the detection range is limited by the size of the aperture. Highly polydisperse samples containing larger particles might lead to a blockage of the pore that makes clearing necessary and, in turn, might cause changes of the pore shape and size. On the other hand, flow cytometry provides limited data on particle size and size distribution, but offers particle quantification and determination of other physical properties. The development of TRPS and better flow cytometers improved the applicability in terms of particle detection in biopharmaceutical research; however, there are still drawbacks, especially in the optical systems.

Emerging principles, like the one underlying suspended microchannels (SMR), open novel possibilities. Thanks to their ability to precisely discriminate between particles of different densities they are able to detect and characterize particle by differences in material or conformation.

Modern microscopic approaches, such as micro-flow imaging (MFI) offer a more automated, high-throughput approach for quantifying as well as optimized imaging tools for a better characterization of particles. Microscopic techniques are, however, constrained by resolution limits. Current common techniques set the limit of the application of optical microscopy to the micrometer size range.

Size and particle size distributions as well as particle concentration are the parameters most often sought in particle analysis, and analytical methods provide this information, after a fashion. Reported particle “size” is generally a spherical approximation. Information about additional properties, such as shape, is needed to fully characterize a suspension and predict its behavior. Developing this information may enable better comparability and cross-correlations between different methods. To date, major challenges remain, despite exciting advances in the field: very high or low concentration suspensions, high polydispersity, and measurement over the whole range of subvisible particle diameters remain difficult or impossible. Among the most challenging samples are biopharmaceuticals (including monoclonal antibodies) and only a few particle-analysis techniques can provide all of the information the discipline demands (Table 2). Further developments of established techniques as well as new approaches, indeed, are essential.

New techniques have emerged over the past decade: nanoparticle tracking analysis, micro flow imaging, tunable resistive pulse sensing, improved analytical centrifuges, and better flow cytometers. Analytical science is making progress, but the need for new, robust, reliable, and better particle-analysis techniques continues, as well as the need to develop stable standard particles that match proteinaceous properties.

## Figures and Tables

**Table 1:** Particle detection and characterization principles.

	Particle Light Interactions	Microscopy and Imaging	Resistive Pulse Sensing	Suspended Microchannel Resonators	Sedimentation and Centrifugation	Further Separation Approaches
**Physical phenomenon**	Absorption, diffraction, refraction, scattering, fluorescence, polarization, etc.	Space-resolved imaging of particles coded by colors and intensity	Current blockage of specific intensity and duration	Change in the resonator frequency density and size dependent	Sedimentation velocity, separation	Separation in fractions due to a particle property
**Output particle properties (examples)**	Size and size distribution, molecular weight, material and structural properties, concentration, interaction parameter	Size and size distribution, shape, optical properties, concentration, etc.	Size and size distribution, shape, concentration, charge, etc.	Size and size distribution, concentration, density, material differentiation, etc.	Size and size distribution, shape, density, equilibrium parameters, etc.	Depending on the investigated property and the used detector
**Main advantage(s)**	Variable approaches possible	Direct measurement and imaging	Independent of optical/material properties	Differentiation between materials	Variable approaches, no destructive preparation expected	Analysis of separated fractions, advantageous for polydisperse/polymodal samples
**Main challenge(s)**	Complex physical processes and calculation, polydispersity	Resolution, preparation	Conductivity, polydispersity	Channel blockage due to interactions	Material properties necessary; no concentration determination	Separation conditions
**Techniques** **(examples)**	LO, SLS, DLS, turbidimetry, UV/Vis spectroscopy and spectrometry, Raman spectroscopy, flow cytometers, etc.	Various types of microscopes, e.g., optical, REM, TEM, AFM, flow imaging devices, e.g., MFI	Coulter counters and some FACS instruments	Chip-based SMRs, commercial instrument: Archimedes	All types of preparative and analytical centrifuges, e.g., AUC, DISC	Chromatographic methods, e.g., HP-SEC; AF4; SDS-PAGE

**Table 2:** Examples of current principles of established and alternative techniques for biopharmaceuticals in comparison to light obscuration.

	Light Obscuration (LO)	Micro- Flow Imaging (MFI)	(Nano-) Particle Tracking Analysis (NTA)	Tunable Resistive Pulse Sensing (TRPS)	Space and Time Resolved Extinction Profile (STEP)	Flow Cytometry (FC)
**Operating principle**	Particle–light interactions (static), particle-by-particle	Microscopy and imaging, particle-by-particle	Particle–light interactions (dyn.), particle-by-particle	Electrozone sensing/Coulter principle, particle-by-particle	Sedimentation and centrifugation, ensemble	Particle–light interactions (static), particle-by-particle
Size range *	1–600 µm	2–100 µm	20–1000 nm	50 nm–10 µm	20 nm–100 µm	100 nm–100 µm
**Input/analysis required information**	No	No	Measurement and evaluation settings	Choose pore size, set stretch, pressure, voltage	RI and density for PSD, centrifugation protocol	Size standard for size estimations, settings
**Output/provided information**						
Particle concentration	Yes	Yes	Yes	Yes	No	Yes
Particle size	Yes	Yes	Yes	Yes	Yes	Limited
Size distribution (PSD)	Yes	Yes	Yes	Yes	Yes	Limited
Shape	-	Circularity, aspect ratio	-	Yes (duration)	-	Estimations
Structure	-	Related to contrast	-	-	-	Estimations
Identity	-	Due to shape/structure	-	-	-	-
Other	-	particle images	Scatter intensities and RI	Particle charge	sedimentation velocities, suspension stability	polydispersity, (physical properties)
**Equivalent particle** **diameter**	Projected area	Projected area	Hydrodynamic	Volume	Volume	Scatter profile
**Material differentiation**	No	Yes; due to shape and gray scale	No; potential differentiation due to scatter intensity	No; potential differentiation due to charge	No	Estimations can be made based on calibration standards
**Detection of translucent particles**	+	++	++	+++	+	++
**Destructive**	No/yes (dilution)	No/yes (dilution)	No	No/yes by voltage	No/yes by crowding	No
**Particle separation required**	No	No	No/yes for highly polydisperse suspensions	No/yes for highly polydisperse suspensions	No	No
**Sample handling and preparation**	++	++	++	++	+++	++
**Sample volume (minimal)**	2 mL/25 mL (Ph.Eur.)	0.5–1.0 mL	0.5–1.0 mL	40–120 µL	0.4–1.5 mL	100–200 µL
**Particle concentration**	≤18,000 counts/mL	≤850,000 counts/mL	10^7^–10^9^ particle/mL	10^5^–10^11^ particle/mL	0.00015–90 Vol %	n/a
**Calibration Yes/No**	No	No	No	Yes	No/yes	No/yes
**Speed/sample**		8 min	12 min	10 min	Depending on sample	1 min
**High throughput/autosampler**	Yes/no autosampler	Yes/autosampler	No/no autosampler	Yes/no autosampler	Yes/12 samples simultaneously	Yes/autosampler
**Provided Software**						
Measurement	Yes	Yes	Yes	Yes	Yes	Yes
Data evaluation	Yes	Yes	Yes	Yes	Yes	Yes
Report	Yes	Yes	Yes	Yes	Yes	-
**Data export**						
Raw data		CSV	CSV, video	CSV,	CSV, PDF	CSV
Graph/processed		PDF	PDF, jpeg	rtf, jpeg	CSV, PDF	Images
**Particle size distribution**						
Accuracy	++	++	++	+++	++	++
Repeatability	++	+++	++	+++	+++	++
Resolution	++	++	++	+++	++	++
**Protein applicability**						
BSA standard	+++	+++	+++	+++	+++	+++
mAB solution	+++	+++	++	+	+	++
**Portability/space**	++	++	+ (NS500), ++ later versions	+++	++	++ (depending on the system)

* limits as mentioned by the manufacturers; depending on the material these limits might vary, e.g., NTA detects gold nanoparticle of 10 nm but starts detection of protein particle of 50 nm.
